# Differences in the Formation of Reactive Oxygen Species and Their Cytotoxicity between Thiols Combined with Aqua- and Cyanocobalamins

**DOI:** 10.3390/ijms231911032

**Published:** 2022-09-20

**Authors:** Yuri V. Shatalin, Victoria S. Shubina, Marina E. Solovieva, Vladimir S. Akatov

**Affiliations:** Institute of Theoretical and Experimental Biophysics, Russian Academy of Sciences, Institutskaya 3, 142290 Pushchino, Russia

**Keywords:** hydroxocobalamin, cyanocobalamin, thiolatocobalamins, thiol oxidation, reactive oxygen species, cytotoxicity

## Abstract

Cobalamin is an essential nutrient required for the normal functioning of cells. Its deficiency can lead to various pathological states. Hydroxocobalamin (HOCbl) and cyanocobalamin (CNCbl) are the forms of vitamin B12 that are most commonly used for supplementation. There is substantial evidence indicating that cobalamins can both suppress and promote oxidative stress; however, the mechanisms underlying these effects are poorly understood. Here, it was shown that the oxidation of thiols catalyzed by HOCbl and CNCbl is accompanied by reactive oxygen species (ROS) production and induces, under certain conditions, oxidative stress and cell death. The form of vitamin B12 and the structure of thiol play a decisive role in these processes. It was found that the mechanisms and kinetics of thiol oxidation catalyzed by HOCbl and CNCbl differ substantially. HOCbl increased the rate of oxidation of thiols to a greater extent than CNCbl, but quenched ROS in combination with certain thiols. Oxidation catalyzed by CNCbl was generally slower. Yet, the absence of ROS quenching resulted in their higher accumulation. The aforementioned results might explain a more pronounced cytotoxicity induced by combinations of thiols with CNCbl. On the whole, the data obtained provide a new insight into the redox processes in which cobalamins are involved. Our results might also be helpful in developing new approaches to the treatment of some cobalamin-responsive disorders in which oxidative stress is an important component.

## 1. Introduction

**Figure 1:**
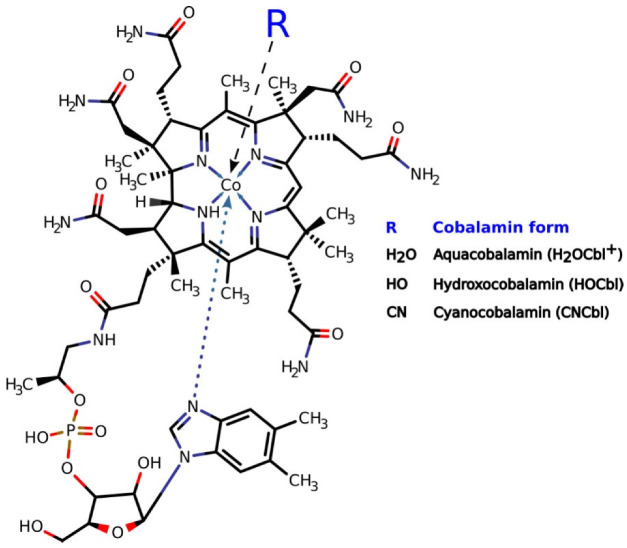
Structure of Vitamin B12.

Vitamin B12 (cobalamin) is a water-soluble vitamin; its presence in human diet is necessary for normal cell function. It exists in several related forms (vitamers). All forms of vitamin B12 consist of a central cobalt ion, an organic component called the corrin ring, and the attached nucleotide base (Figure 1). The corrin ring has four pyrrole moieties. Two of them are directly bonded to each other, whereas the other pyrrole groups are linked through a methene bridge. The cobalt ion is bonded to four pyrrole nitrogen atoms and can form additional bonds on the lower and upper surfaces of the corrin ring (axial positions). The formation of a coordination bond at these positions depends on the redox state of the cobalt ion and environmental conditions. Thus, [Co^1+^]Cbl is predominantly four-coordinate and has no axial ligands. [Co^2+^]Cbl and [Co^3+^]Cbl are typically five- and six-coordinate, with one and two axial ligands, respectively. In free cobalamin, the lower axial position (α-position) is often occupied by a nitrogen atom of 5,6-dimethylbenzimidazole (Bzm), which is covalently bound to the corrin ring via an intramolecular loop. However, the Co-Bzm bond is not strong, and the base can dissociate depending on pH, temperature, and environment [1,2,3]. The cobalt-bound and cobalt-dissociated conformations of Bzm are called base-on and base-off, respectively. The upper axial position (β-position) can be occupied by cyanide (CN^−^), water (H_2_O or HO^−^ depending on pH), 5′-deoxyadenosine, or a methyl group, as well as some other ligands. There are also structural analogues of cobalamins that lack the dimethylbenzimidazole ribonucleotide group (cobinamides).

In mammals, methylcobalamin and adenosylcobalamin are the cofactors of metabolically important enzymes methionine synthase and methylmalonyl-CoA (MM-CoA) mutase, respectively. The cytosolic methionine synthase catalyzes the transfer of a methyl group to homocysteine from N5-methyltetrahydrofolate. As a result, methionine and tetrahydrofolate are formed. The latter plays an important role in the metabolism of amino acids and nucleotides. In particular, it participates in the synthesis of purines and one pyrimidine of thymine, which are needed for the synthesis of nucleic acids. Methylmalonyl-CoA mutase is a mitochondrial matrix enzyme, which catalyzes the isomerization of L-methylmalonyl-CoA into succinyl-CoA, an important reaction in the metabolism of amino acids and fatty acids. Succinyl CoA is also required for the synthesis of the heme and, hence, of hemoglobin.

A deficiency of vitamin B12 can lead to various pathological states, including neurological, psychiatric, and hematological disorders. It is usually caused by the malabsorption of the vitamin or its inadequate dietary intake. There are also genetic defects leading to the impairment of the functioning of cobalamin-dependent enzymes and proteins involved in the transport and metabolism of cobalamin. In the latter case, the deficiency of cobalamin also causes some reduction in the activities of methionine synthase and methylmalonyl-CoA mutase. In most cases, the treatment of vitamin B12 deficiency involves the injection or ingestion of large quantities of hydroxolcobalamin (HOCbl) and cyanocobalamin (CNCbl) [4,5,6]. HOCbl is a naturally occurring intracellular form of cobalamin, whereas CNCbl is a synthetic form. The latter vitamin B12 form was isolated first and received wide recognition in the treatment of vitamin B12 deficiency [6]. The low cost and stability of CNCbl contribute to its wide use as a dietary supplement and an additive for food fortification. Yet, in some countries, HOCbl has completely replaced CNCbl as a drug of first choice for vitamin B12 replacement therapy [5,6], because it is retained in the body longer and can be administered at longer intervals than CNCbl [5]. In addition, HOCbl tightly binds cyanide and is used as an antidote to cyanide poisoning [4,5]. In this case, HOCbl is administered at very high doses (5–10 (g) intravenously [7]), which significantly elevates its concentration in the blood serum [7,8]. In some cases, maximal concentration of free HOCbl can reach hundreds of micromoles (267–1011 μM [8]) vs. 0.17–0.92 nM of the protein-bound Cbl under the normal circumstances. This leads to the formation of non-toxic CNCbl, largely excreted to urine [4].

Nowadays, the main pathways of absorption, transport, internalization, and metabolism of cobalamins have been clarified [9,10]. We should mention that the interaction of cobalamins with high- and low-molecular-weight sulfur-containing compounds plays an important role in the enzymatic activation of inert vitamins, as well as their redox and coordination chemistry [9,11,12,13,14]. In particular, glutathion (GSH), the most abundant low-molecular-weight thiol in cells [15], is involved in cobalamin metabolism. Glutathionylcobalamin (GSCbl) is an intermediate that is temporarily formed in cells and, probably, represents a precursor of de novo synthesized and enzyme-bound MeCbl and AdoCbl [16,17,18]. GSH is involved in the dealkylation of AdoCbl and MeCbl, as well as, under anaerobic condition, in the decyanation of CNCbl and the removal of the OH group in HOCbl mediated by CblC-protein (CblC). Defects in CblC (*cblC* gene) result in the impaired conversion of dietary vitamin B12 to its coenzyme forms (AdoCbl and MeCbl) [19,20]. A functional deficiency of MeCbl and AdoCbl leads to a significant deactivation of cobalamin-dependent enzymes and the accumulation of homocysteine and methylmalonic acid. CNCbl is inefficient in the treatment of patients with cobalamin C disease [21,22], whereas HOCbl at high doses has a positive effect [21,22,23,24,25,26]. It is important that, in some patients with a CblC failure or/and problems with the specific cellular Cbl-uptake, the concentration of HOCbl in the serum should approach the micromolar level to attain an optimal metabolic response [25,27,28]. The treatment can last for several months [25,29]. In turn, the safety of a long-term therapy with high-dose HOCbl has not been adequately examined [27].

The advantages of HOCbl therapy in comparison to CNCbl are not fully understood. According to one suggestion, the exposed nature of the Co ion in HOCbl facilitates its nonspecific chemical reduction to [Co^2+^]Cbl, which can be subsequently converted to the coenzyme forms [1]. GSH is considered as a potential reducing agent [1]. However, it should be noted that the oxidation of thiols by cobalamins is accompanied by ROS production and may induce oxidative stress. In particular, the formation of hydrogen peroxide during the cobalamin-catalyzed oxidation of 2-mercaptoethanol and dithioerythritol has been reported [30]. We have previously shown that HOCbl in combination with thiols such as GSH, N-acetylcysteine (NAC), and dithiothreitol (DTT) catalyzes the formation of hydrogen peroxide in culture medium, leading to cell death [31,32]. Catalase completely prevents oxidative stress and cell death induced by combinations of HOCbl with these thiols, demonstrating that H_2_O_2_ plays an important role in these processes. We hypothesize that the oxidation of thiols by cobalamins, accompanied by ROS production, contributes to the development of oxidative stress in the CblC disease. For example, the pathological variants of CblC (R161G/Q) exhibit thiol oxidase activity, which is suppressed in wild-type human CblC. These mutants oxidize GSH to GSSG and reduce dissolved oxygen to superoxide anions [33,34]. Taking into account the aforesaid, it may be assumed that a defective CblC-Cbl complex, as well as the excessive free HOCbl accumulated during the treatment of CblC patients, might spontaneously oxidize GSH. In both cases, the redox process is accompanied by ROS production and may contribute to oxidative stress. In addition, the patients with CblC deficiency show a depletion of cysteine, a thiol-containing amino acid [35]. This may also be due to direct interaction of thiol with cobalamin [35].

On the other hand, the available literature indicates that combinations of thiols (GSH and NAC) with standard cobalamin derivatives (HOCbl, MeCbl, CNCbl) protect cells against H_2_O_2_-induced oxidative stress [36]. At the same time, the thiolatocobalamin complexes GSCbl and NACCbl taken alone demonstrate significantly greater protective capabilities than the combinations mentioned above or the thiols alone. In particular, thiolatocobalamins are considered as a possible alternative to HOCbl in the treatment of patients with the *cblC* disease [11,35].

It remains unclear how different forms of vitamin B12 affect the development/suppression of oxidative stress. The key modulators of cell survival and death (such as products of the cobalamin-catalyzed oxidation) also remain unknown. Our previous results indicate that combinations of HOCbl with GSH, NAC, DTT, and ascorbic acid (AA) lead to apoptotic death in cancer cells [31,32,37]. At the same time, a combination of HOCbl with sodium diethyldithiocarbamate (DDC) leads to entosis [38] and/or paraptosis-like cell death [38,39]. For a more complete understanding of mechanisms underlying the effects of cobalamin-reducing agent combinations, a deeper insight into ongoing chemical processes is needed. The aim of the present work was a comparative study of the interaction of HOCbl and CNCbl with some low-molecular-weight thiols, as well as a comparative analysis of ROS production during the cobalamin-catalyzed oxidation of thiols and of the cytotoxic effects of thiol–cobalamin combinations.

## 2. Results

Under physiological conditions, HOCbl is partially converted to H_2_OCbl^+^ (H_2_OCbl^+^/HOCbl, pKa ≈ 7.8 [40]). As a result, an equilibrium between these two forms (HOCbl and H_2_OCbl^+^) is established. Therefore, the solutions of HOCbl in PBS (pH 7.2) are subsequently designated as H_2_OCbl^+^/HOCbl. It should be noted that, as opposed to HOCbl, the β-axial ligand in H_2_OCbl^+^ can be readily replaced by other ligands [11,40,41,42,43,44], indicating that H_2_OCbl^+^ enters into the reaction with thiols.

### 2.1. ROS Production during the Oxidation of Thiols Catalyzed by H_2_OCbl^+^/HOCbl and CNCbl

**Figure 2:**
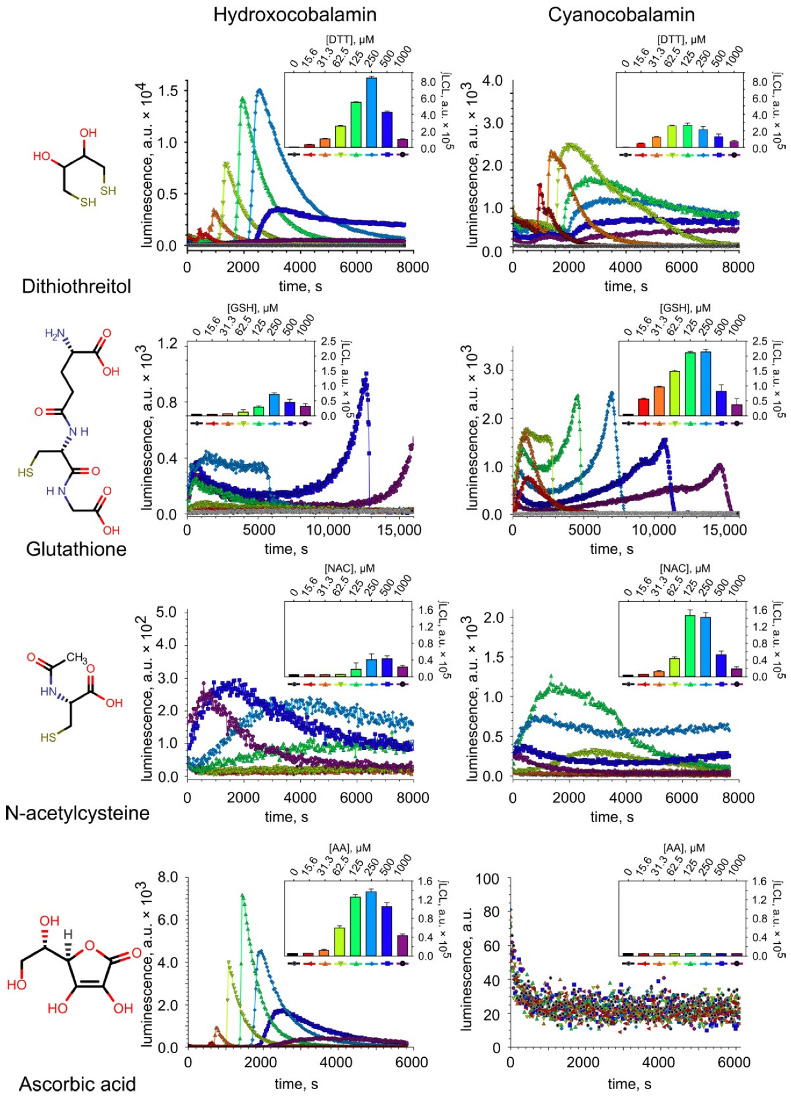
Chemiluminescence recorded during the oxidation of compounds tested in the presence of cobalamins. Inserts: Dependence of integral chemiluminescence response (∫LCL) on the concentration of the compounds.

To detect ROS formed during the cobalamin-catalyzed oxidation of sulfur-containing compounds, the method of LCL was used for the first time. It was found that the oxidation of thiols (GSH, NAC, DTT) by cobalamins (H_2_OCbl^+^/HOCbl and CNCbl) gives rise to the emission of chemiluminescence, indicating the formation of ROS in the system (Figure 2, Table S1). An increase in the concentration of thiols caused an increase in the integral LCL response to some maximal value (∫LCLmax) (Figure 2, inserts). A further increase in the concentration of thiols led to a decrease in integral LCL response, which may be due to the antioxidant properties of these compounds (Figure S1). A comparison of ∫LCLmax values (H_2_OCbl^+^/HOCbl vs. CNCbl) indicates that the oxidation of DTT catalyzed by H_2_OCbl^+^/HOCbl results in a higher LCL response than the oxidation of this dithiol catalyzed by CNCbl (Figure 2). In contrast, the oxidation of the monothiols GSH and NAC catalyzed by H_2_OCbl^+^/HOCbl leads to lower LCL responses than the oxidation of these compounds catalyzed by CNCbl (Figure 2). These data are treated in detail in the Discussion section. Among reactions catalyzed by H_2_OCbl^+^/HOCbl, the highest LCL response is observed during the oxidation of the dithiol DTT, whereas the oxidation of monothiols GSH and NAC results in lower LCL responses. Among CNCbl-catalyzed reactions, the oxidation of the dithiol DTT and monothiol GSH leads to comparable LCL responses, whereas the oxidation of NAC results in the lowest LCL response. 

In systems containing either of the vitamin B12 forms and DDC, LCL response was not detected (Figure S2). In systems containing AA, which was used in the study as a standard reducing agent, a high LCL response was observed only in the presence of H_2_OCbl^+^/HOCbl (Figure 2). In the presence of CNCbl, LCL was not detected in the concentration range used, which may be due to both the antioxidant properties of AA and the slow rate of its oxidation.

### 2.2. Kinetics of Thiol Oxidation Catalyzed by H_2_OCbl^+^/HOCbl and CNCbl

**Figure 3:**
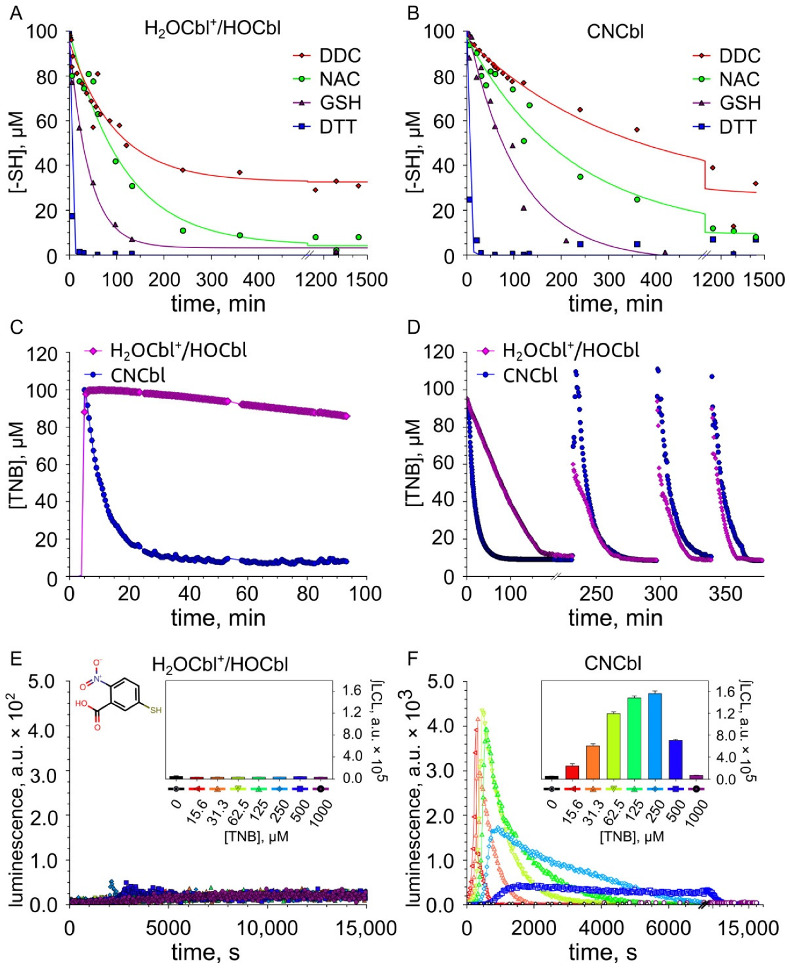
The oxidation of thiols in the presence of cobalamins. (**A**,**B**) Changes in the concentrations of thiol-containing compounds in the presence of cobalamins. (**C**) Changes in the concentration of TNB formed in the reaction of DTNB with NAC in the presence of cobalamins. (**D**) Changes in the concentration of TNB preliminarily formed in the reaction of DTNB with DTT in the presence of cobalamins during four catalytic cycles. (**E**,**F**) Chemiluminescence recorded during the oxidation of TNB in the presence of cobalamins. Inserts: Dependence of integral chemiluminescence response (∫LCL) on the concentration of TNB.

The concentration of thiols in the presence of different forms of vitamin B12 was evaluated using the Ellman’s reagent (DTNB). Changes in the concentrations of thiols in the presence of cobalamins are shown in Figure 3. 

It is seen that the oxidation of thiols DTT, GSH, and NAC catalyzed by H_2_OCbl^+^/HOCbl proceeds at a higher rate than their oxidation by CNCbl (Figure 3A,B). Both H_2_OCbl^+^/HOCbl and CNCbl catalyze the oxidation of dithiol DTT at a higher rate than the oxidation of GSH (Table 1). In turn, the oxidation of GSH occurs at a higher rate than the oxidation of NAC.

The addition of DTNB to a mixture containing NAC and H_2_OCbl^+^/HOCbl leads to a fast formation of 2-nitro-5-thiobenzoate (TNB), as indicated by the appearance of the characteristic absorption band with a maximum at 412 nm. Then, a slow oxidation of TNB occurs (the intensity of absorption at 412 nm decreases) (Figure 3C). The pseudo-first-order rate constant for this reaction (k_1_) is 3.24 × 10^−5^ s^−1^. On the other hand, the oxidation of TNB, which was preliminarily obtained in the reaction of DTNB with DTT, occurs at a somewhat higher rate (Figure 3D). The pseudo-first-order rate constant for the oxidation of TNB under these conditions (k_2_) is 6.42 × 10^−5^ s^−1^. In turn, the addition of DTNB to a mixture containing NAC and CNCbl, as in the case of H_2_OCbl^+^/HOCbl, results in a fast formation of TNB. However, the oxidation of TNB catalyzed by CNCbl proceeds at a much higher rate than its oxidation by H_2_OCbl^+^/HOCbl (Figure 3C). The pseudo-first-order rate constant for this reaction catalyzed by CNCbl (k_3_) is 2.42 × 10^−3^ s^−1^. The CNCbl-catalyzed oxidation of TNB pre-formed during the reaction of DTNB with DTT occurs at a somewhat lower rate with a rate constant (k_4_) of 1.02 × 10^−3^ s^−1^ (Figure 3D).

Thus, it was shown for the first time that both forms of vitamin B12 catalyze the oxidation of TNB, which is formed during the reduction of DTNB by thiols. Meanwhile, CNCbl catalyzes the oxidation of TNB at a much higher rate than H_2_OCbl^+^/HOCbl (Figure 3C,D).

After complete oxidation of TNB, the addition of a new aliquot of DTT results in the regeneration of TNB, after which the oxidation of this thiol occurs again (Figure 3D). It was found that the rate of TNB oxidation in the presence of H_2_OCbl^+^/HOCbl increases more than 15 times after the first catalytic cycle. However, the subsequent increase in the rate is not so significant. The rate of TNB oxidation in the fourth cycle is only two times higher than that in the second cycle. The rate of CNCbl-catalyzed oxidation of TNB slightly increases with each catalytic cycle. The rate of TNB oxidation in the fourth cycle is only 1.37 times higher than that in the first cycle. As a result, in the fourth cycle, the rate of oxidation of TNB catalyzed by H_2_OCbl^+^/HOCbl begins to exceed the rate of CNCbl-catalyzed oxidation of this thiol (Figure 3D, Table 1). 

It is significant that, during the first cycle of TNB oxidation, a high CL response was observed only in the presence of CNCbl, whereas in the presence of H_2_OCbl^+^/HOCbl no CL was detected (Figure 3E,F).

### 2.3. Examination of the Reaction between Cobalamins and Thiols by UV-Visible Spectroscopy

Cobalamins absorb light in the UV-visible region of the spectrum. Characteristic bands of the spectra of “typical” cobalamins are designated by α/β (420–600 nm), γ (350–420 nm), and δ (300–330 nm) [45]. The thiols GSH, NAC, and DTT do not absorb light in the wavelength range of 210–700 nm. Therefore, in the wavelength range used in the experiment, the spectral changes of the solutions containing cobalamin and one of the above-mentioned thiols can be assigned to electronic transitions in the structure of cobalamin. 

Our data demonstrate that HOCbl is predominantly converted to H_2_OCbl^+^ at physiological pH (maximum absorbance at 350, 412, and 525 nm [40,43,46,47]) (Figure S3).

**Figure 4:**
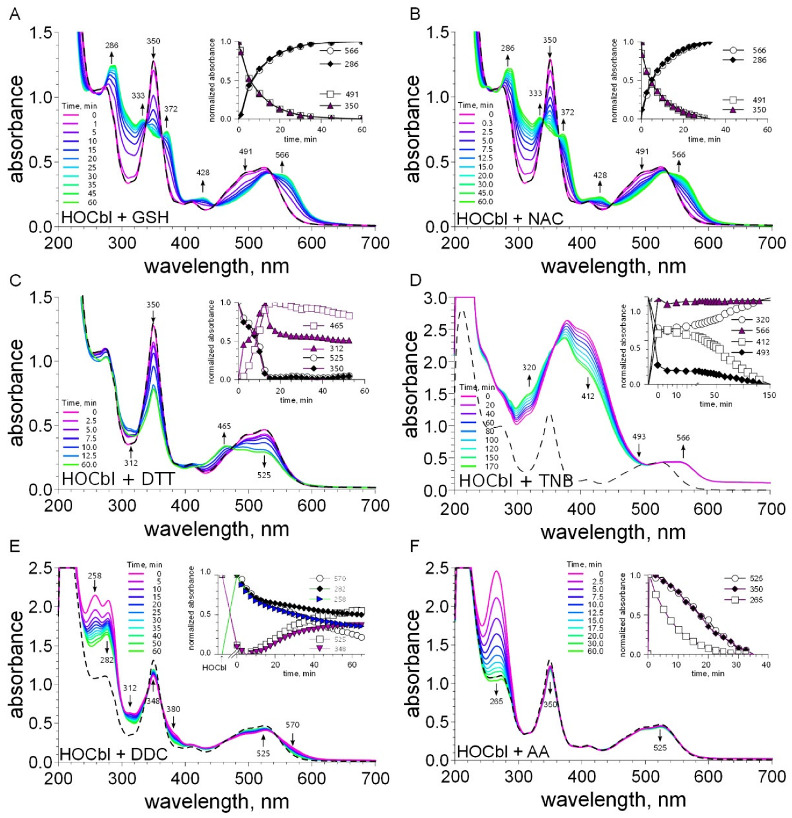
UV-Vis spectra recorded in the reactions between H_2_OCbl^+^/HOCbl and compounds being tested: (**A**) GSH, (**B**) NAC, (**C**) DTT, (**D**) TNB, (**E**) DDC, (**F**) AA. Dashed lines indicate the spectrum of H_2_OCbl^+^/HOCbl. Inserts: kinetic traces recorded in the corresponding reactions at selected wavelengths.

The addition of a twofold excess of GSH to H_2_OCbl^+^/HOCbl leads to changes in absorption spectra with time (Figure 4A). 

In particular, we found a decrease in absorbance at 350 (disappearance of aquacobalamin [11]) and 491 nm, as well as an increase in absorbance and the appearance of new peaks at 333, 372, 428, and 566 nm (formation of thiolatocobalamin [11,43,48,49]). Isosbestic points were found at 338, 363, 446, and 536 nm (Figure S4A). Similar spectral changes were observed in a solution containing H_2_OCbl^+^/HOCbl and NAC (Figure 4B and Figure S5A). Thus, the data obtained indicate the replacement of coordinated water in aquacobalamin by thiols and the formation of thiolatocobalamin complexes [11,43,48,49].

The addition of a twofold excess of DTT to H_2_OCbl^+^/HOCbl did not lead to any significant changes in absorption spectra with time. However, in the presence of a tenfold excess of this dithiol, the spectrum underwent pronounced changes (Figure 4C and Figure S6A). An increase in absorbance at 465 and 312 nm was accompanied by a decrease in absorbance at 350 and 525 nm (disappearance of aquacobalamin). It may be assumed that these spectral changes result from the formation of a complex of cobalamin with DTT or/and the reduction of Cbl(III) to Cbl(II). Twelve to seventeen minutes after the start of the reaction, a decrease in absorbance at 465 and 312 nm, as well as an increase in absorbance at 525 nm, were observed (Figure 4C, insert), suggesting the oxidation of Co(II) to Co(III) or/and the decomposition of existing complexes.

The UV-Vis spectra of DDC, TNB, and AA overlapped to some extent with those of cobalamins. Therefore, the spectral changes of the mixtures of cobalamin with DDC, TNB, or AA can be assigned to both electronic transitions in the cobalamin structure and structural changes in these compounds. Figure 4D shows spectral changes observed during the reaction between H_2_OCbl^+^/HOCbl and TNB. TNB absorbs light at 412 nm. Immediately after the addition of a twofold excess of TNB to a H_2_OCbl^+^/HOCbl solution, an increase in absorbance at this wavelength occurred (attributed to the overlapping of the bands of TNB and H_2_OCbl^+^/HOCbl). In addition, the absorbance at 566 nm increased, suggesting the formation of a thiolatocobalamin complex. The time-dependent spectra showed a decrease in the absorbance at 412 nm and an increase in the absorbance at 320 nm (Figure 4D and Figure S7A), which indicates that the oxidation of TNB leads to the formation of DTNB. Isosbestic points were observed at 268 and 356 nm (Figure S7A). 

DDC absorbs light at 206, 258, and 282 nm. Immediately after the addition of a twofold excess of DDC to a H_2_OCbl^+^/HOCbl solution, the absorption spectra of the mixture showed changes: an increase in absorbance at 258, 282 (attributed to the overlapping of bands of DDC and H_2_OCbl^+^/HOCbl), 312, 380, and 570 nm, as well as a decrease in absorbance at 348, 491, and 525 (Figure 4E and Figure S8A). These data suggest the replacement of coordinated water in aquacobalamin (a decrease in absorbance at 348 nm [11]) and, probably, the formation of a cobalamin–DDC complex. Within the next 5 min, the absorbance at 258 and 282 nm decreased, indicating the oxidation of DDC. At the same time, a further minor increase in absorbance at 312 nm and a decrease in absorbance at 348 nm were observed. About 5–7 min after the start of the reaction, absorbance at 258, 282, 312, 380, and 570 nm decreased, and it increased at 348, 491, and 525 nm. It may be assumed that, during the oxidation of DDC, a transformation of the cobalamin–DDC complex occurs.

AA also absorbs light in the UV region with a maximum at 265 nm. An increase in absorbance at this wavelength, attributed to the overlapping of bands of AA and H_2_OCbl^+^/HOCbl, was observed immediately after the addition of AA to H_2_OCbl^+^/HOCbl (Figure 4F). The time-dependent spectra showed a rapid decrease in the absorbance at 265 nm, indicating the oxidation of AA. Minor changes in the regions that correspond to the absorption maxima of H_2_OCbl^+^ (γ, α/β) were observed. The difference absorption spectra showed a decrease in the absorbance at 350 and 525 nm and an increase in the absorbance at 442 nm (Figure S9A). These data suggest that the oxidation of AA proceeds via the formation of the unstable intermediate aquacobalamin-ascorbate. 

**Figure 5:**
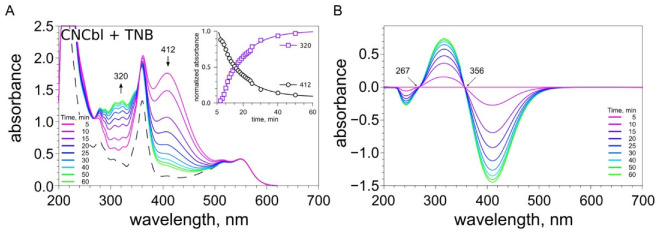
UV-Vis spectra recorded in the reactions between CNCbl and TNB. (**A**) Absorption spectra. Insert: kinetic traces recorded in the corresponding reaction at selected wavelengths. (**B**) Difference spectra.

After the addition of GSH, NAC, or DTT to a solution of CNCbl, no significant changes in the absorption spectrum were observed (Figures S4B–6B). In cases where the absorption bands of CNCbl and a compound being tested (TNB, DDC, or AA) overlapped, spectral changes were observed in the regions corresponding to this very compound (Figures S7B–9B). As an example, Figure 5 shows time-dependent changes in the absorption of a solution containing CNCbl and TNB. It is seen that absorbance at 412 nm (assigned to TNB) decreases, whereas the absorbance at 320 nm (assigned to DTNB) increases. These changes were accompanied by the appearance of the isosbestic point at 356 nm (Figure 5B). These data indicate that TNB is oxidized to DTNB. Any significant changes in the regions that correspond to the absorption maxima of CNCbl were not observed (γ, α/β). In the whole, the data obtained suggest that, during the reactions of CNCbl with compounds being tested, the replacement of the CN-group in CNCbl does not occur. 

### 2.4. A Comparative Examination of the Cytotoxic Effect of the Combinations of Thiols with H_2_OCbl^+^/HOCbl and CNCbl

**Figure 6:**
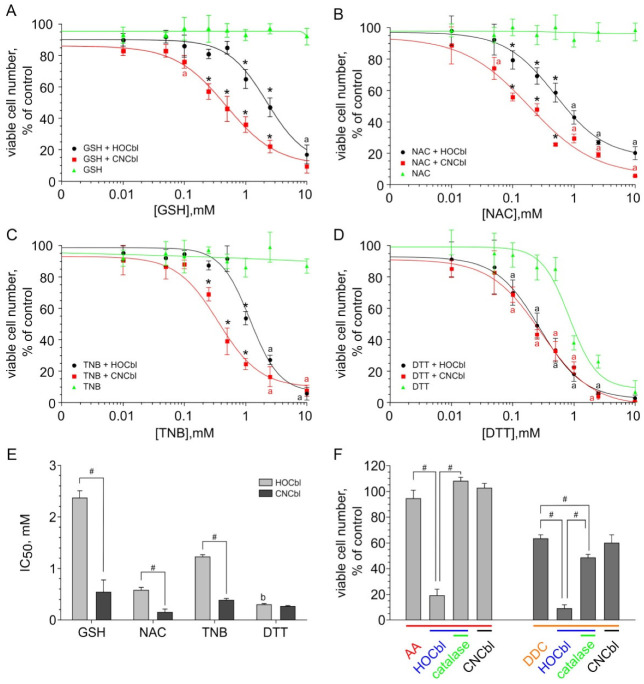
Cytotoxic effect of combinations of cobalamins with thiols (**A**–**D**), IC50 values for combinations of cobalamins with thiols (**E**), and cytotoxicity of combinations of cobalamins (25 μM) with AA (0.5 mM) and DDC (1 mM) (**F**). The results were expressed as the mean ± standard deviation. Data were analyzed using one-way ANOVA followed by Tukeyʹs test. (**A**–**D**) * Differences are significant compared with other two groups; ^a^ significant differences between the cobalamin+thiol and thiol groups. (**E**,**F**) ^#^ significant differences between the groups; ^b^ differences are significant compared with combinations HOCbl + monothiols, *p* < 0.05.

It was shown that GSH, NAC, and TNB added in the concentration range used (up to 10 mM) had no cytotoxic effect on MCF7 cells, in contrast to DTT. The IC50 value (the concentration of a substance at which the number of living cells is reduced by half compared to the control) of DTT administered alone was 0.93 ± 0.04 mM. At the same time, a combination of monothiols with B12 derivatives produced a cytotoxic effect (Figure 6A–C). A combination of DDT with the cobalamins significantly enhanced its cytotoxicity (Figure 6D). The cytotoxic effect of the thiols combined with cobalamins was associated with cell death. The percent of dead cells was 70–90% at concentrations of thiols 2–3 mM if they were added with the cobalamins. The IC50 values for thiols in combination with 25 μM cobalamins are presented in Figure 6E. It is seen that all monothiols being tested in combination with CNCbl exhibited a more pronounced cytotoxic effect than in combination with H_2_OCbl^+^/HOCbl. There were no significant differences between combinations DTT + CNCbl and DTT + H_2_OCbl^+^/HOCbl. 

A comparison of the cytotoxic action of thiols in the presence of H_2_OCbl^+^/HOCbl indicates that the dithiol DTT is more cytotoxic than the monothiols. Among the monothiols, NAC produced the highest cytotoxic effect, whereas GSH exhibited the lowest cytotoxicity. In the presence of CNCbl, there was no significant difference between the cytotoxic effects of the dithiol DDT and the monothiols. We did not observe any significant difference in cytotoxicity between the monothiols. Catalase (100 U/mL) completely prevented the cytotoxic effect induced or enhanced by the addition of cobalamins to thiols, suggesting that the generation of hydrogen peroxide plays a key role in the cytotoxic activity of these combinations. 

DDC, when added alone at a concentration of 1 mM, decreased the number of viable cells to 60–70% of the control with no increase in the percent of dead cells (cytostatic effect). The combination of DDC with H_2_OCbl^+^/HOCbl resulted in the enhancement of its cytotoxicity, decreasing the number of viable cells from 65 ± 5% to 10 ± 5% compared to the control (Figure 6F) and increasing the percent of dead cells in the culture from 5 ± 2% to 85 ± 7%. Catalase only partially inhibited the cytotoxic effect of this combination, which indicates two mechanisms in the induction of cell death by DDC + H_2_OCbl^+^/HOCbl, one of which depends and the other does not depend on the generation of hydrogen peroxide. CNCbl did not enhance the cytotoxicity of DDC (Figure 6F).

AA had no cytotoxic effect when administrated alone (0.5 mM) or in combination with CNCbl (25 µM), whereas a combination of AA with H_2_OCbl^+^/HOCbl led to a significant decrease in the number of live cells compared to non-treated control (15–20%) (Figure 6F) and an increase in the percent of dead cells in culture up to 80–90%. Catalase completely prevented the cell death induced by this combination. Similar results were obtained for HEp2 cells (Figure S10).

Thus, CNCbl was more effective than H_2_OCbl^+^/HOCbl in enhancing the cytotoxicity of GSH, NAC, and TNB, and, conversely, H_2_OCbl^+^/HOCbl was able to enhance the cytotoxicity of AA and DDC, unlike CNCbl. The ability of CNCbl and H_2_OCbl^+^/HOCbl to enhance the cytotoxicity of dithiol DTT was the same.

## 3. Discussion

### 3.1. Proposed Mechanisms for the Oxidation of Thiols Catalyzed by Cobalamins

The results of the study indicate that the oxidation of thiols (GSH, NAC, or DTT) catalyzed by both H_2_OCbl^+^/HOCbl and CNCbl is accompanied by the formation of ROS. The formation of ROS during the oxidation of thiols by cobalamins and cobinamides has been earlier reported in the literature [30,50]. In particular, it was shown that the aerobic oxidation of 2-mercaptoethanol and dithioerythritol by corrinoids results in the formation of hydrogen peroxide and the disulfides of these thiols [30]. In addition, the aquacobalamin-catalyzed oxidation of 2-mercaptoethanol led to the formation of superoxide anions [50]. We have previously shown that thiols GSH, NAC, and DTT in combination with H_2_OCbl^+^/HOCbl are capable of catalyzing the formation of hydrogen peroxide in culture medium [31,32]. The corrinoid-catalyzed oxidation of thiols in these studies was examined by polarography. This method makes it possible to monitor the consumption of oxygen during the reaction and to determine the amount of hydrogen peroxide accumulated in the system by a particular time (determined from an increase in the oxygen concentration in a solution after the addition of catalase). In the frame of the present work, for the first time, the oxidation of thiols catalyzed by cobalamins was studied by luminol-dependent chemiluminescence. This method was previously applied to determine the content of vitamin B12 in pharmaceutical preparations [51,52] and serum [53]. Here, it was used to detect time-dependent changes in ROS production. This enabled us to estimate and compare the total level of ROS formed during thiol oxidation catalyzed by different forms of vitamin B12.

It was found that the oxidation of dithiol DTT in the presence of H_2_OCbl^+^/HOCbl results in a higher ROS production than in the presence of CNCbl. In contrast, the oxidation of monothiols GSH, NAC, and TNB led to a higher ROS production in the presence of CNCbl than in the presence of H_2_OCbl^+^/HOCbl. As it might appear, these results contradict the data given in Figure 3A,B and the literature data, according to which the rate of the monothiol oxidation is higher in the presence of H_2_OCbl^+^/HOCbl [30,54,55]. However, the difference in the level of ROS detected during the H_2_OCbl^+^/HOCbl- and CNCbl-catalyzed oxidation of thiols may be due to differences in the mechanisms of their oxidation by cobalamins or/and in the kinetics of ROS formation and elimination. In the systems containing either of the vitamin B12 forms combined with DDC, ROS production was not being detected. ROS production during the oxidation of ascorbic acid was observed only in the presence of H_2_OCbl^+^/HOCbl. These data suggest that cobalamins combined with some compounds can induce oxidative stress. For combinations of monothiols with cobalamins, the development of oxidative stress is more likely in the presence of CNCbl than in the presence of H_2_OCbl^+^/HOCbl. 

**Figure 7:**
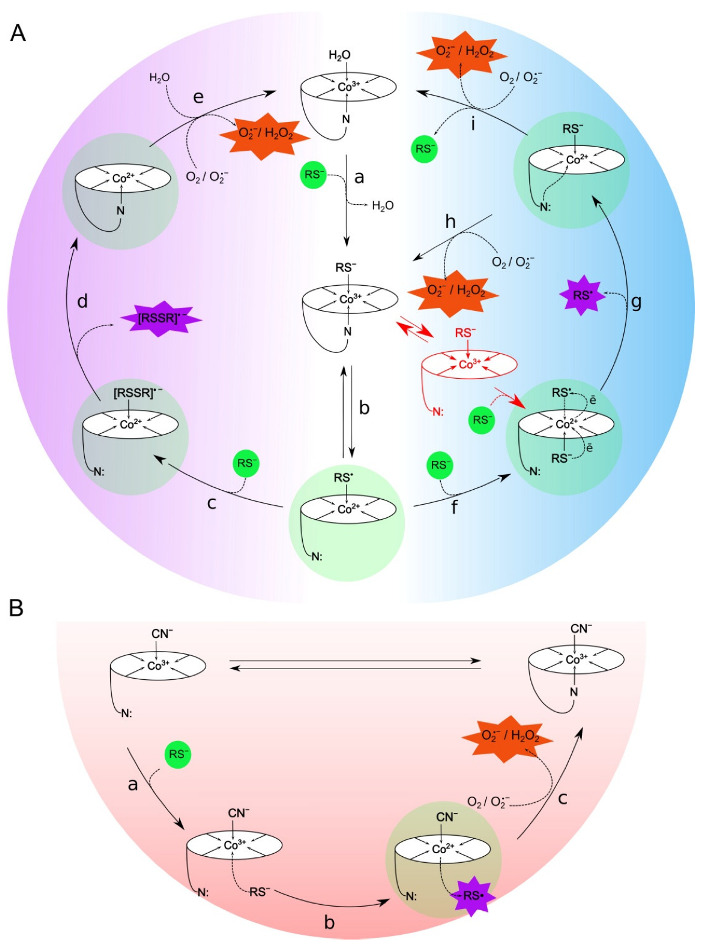
The proposed mechanism for the oxidation of thiols catalyzed by cobalamins. (**A**) H_2_OCbl^+^/HOCbl. The structure shown in red is a possible intermediate formed during the reaction [49]. (**B**) CNCbl. GSH, NAC, and DTT predominantly occur in the protonated form (RSH) at physiological pH. When thiol coordinates to cobalamin, deprotonation and complex formation between RS^−^ and Cbl occur. TNB predominantly exists in the anionic form (RS^−^) at physiological pH.

The data on changes in the concentrations of test compounds in the presence of cobalamins showed that H_2_OCbl^+^/HOCbl catalyzes the oxidation of GSH, NAC, DTT, DDC, and AA at a higher rate than CNCbl. On the other hand, it was found for the first time that TNB in the presence of CNCbl is oxidized faster than in the presence of H_2_OCbl^+^/HOCbl, indicating that the pathways by which thiols are oxidized by CNCbl and H_2_OCbl^+^/HOCbl differ substantially. The available literature data demonstrate that coordinated water in aquacobalamin can easily be replaced by sulfur-containing ligands [11,40,41,42,43,44]. Those are coordinated to the cobalt atom in the β-axial position via the sulfur atom. Our results indicate that the oxidation of thiols in the presence of H_2_OCbl^+^/HOCbl proceeds via the formation of thiolatocobalamin complexes. Based on these findings and the data presented in the literature, the following mechanism of the oxidation of thiols by H_2_OCbl^+^/HOCbl can be proposed (Figure 7A).

Probably, after the replacement of H_2_O by thiolate anion (Figure 7A, step a), intramolecular transfer of the electron from RS^−^ to Co(III) ion and the dissociation of the 5,6-dimethylbenzimidazole nucleotide occur (Figure 7A, step b). Thereafter, the intermediate formed interacts with the second molecule of thiol (or thiolate anion) (Figure 7). Presumably, this process proceeds via two main pathways. The first route involves the formation of the intermediate Cbl(II)RSSR^•−^ (Figure 7A, step c). The formation of similar structures is supported by the data available in the literature [41]. Subsequent release of RSSR^•−^ (Figure 7A, step d) and the oxidation of Co(II) by dissolved oxygen (Figure 7A, step e) lead to the formation of superoxide anions and the regeneration of aquacob(III)alamin in the system. It is also probable that the oxidation of thiols can proceed through the formation of [Co^1+^]Cbl [50]. Then, this intermediate can react with oxygen, resulting in the formation of hydrogen peroxide, or with [Co^3+^]Cbl, giving [Co^2+^]Cbl. The latter is oxidized by oxygen. However, direct evidence for the formation of [Co^1+^]Cbl during the oxidation of thiols is not available [49,50]. The second route involves the coordination of the second molecule of thiol at the α-position and subsequent electron transfer (Figure 7A, step f). This, probably, results in the formation of an intermediate, in which a thiolate anion is coordinated in the β-position and thiyl radical is coordinated in the α-position. The formation of such complexes during the reaction between HOCbl and GSH under anaerobic conditions has been reported earlier by Ramasamy and co-authors [49]. Then, the thiyl radical is released from the α-position of the complex, yielding thiolatocob(II)alamin (Figure 7A, step g). The next step, probably, also proceeds via two routes. The first route involves the oxidation of Co(II) by dissolved oxygen, which leads to the formation of superoxide anions and the regeneration of thiolatocob(III)alamin in the system (Figure 7A, step h). The second route leads to the release of the thiyl radical and the formation of cob(II)alamin, which is subsequently oxidized by oxygen to cob(III)alamin (Figure 7A, step i).

Cyanocobalamin is a stable form of vitamin B12. A high stability constant for the binding of cyanide to aquacobalamin (10^12^ M^−1^ [56]) suggests that the dissociation of cyanide during thiol oxidation is unlikely. In turn, in a solution containing CNCbl as well as other forms of vitamin B12, an equilibrium between base-on and base-off forms is established. In the base-on form, Bzm is coordinated to cobalt in the α-axial position, inhibiting the binding of a ligand to this site. The intramolecular dissociation of the Bzm ligand leads to the formation of base-off cobalamin, in which the α-axial position is accessible to a ligand. The pKa of base-off cyanocobalamin is 0.1, whereas the pKa of base-off aquacobalamin is −2.13 [57]. Therefore, the amount of base-off CNCbl is larger than that of base-off H_2_OCbl^+^. Thus, it can be suggested that the ligand is coordinated to Co in the α-axial position in base-off CNCbl, after which electron transfer from the ligand to Co(III) ions becomes possible. This suggestion is supported by the literature data [58,59]. Spectrophotometric data obtained in the frame of this work indicate that, during the oxidation of thiols by CNCbl, the replacement of the CN-group does not occur. However, the difference absorption spectra showed minor changes at 350 nm during the CNCbl-catalyzed oxidation of DTT, suggesting a weakening of the Co(II)-CN bond (Figure S6B).

The following mechanism of the CNCbl-catalyzed oxidation of thiols can be proposed (Figure 7B). The first step of this process is a coordination of the molecule of thiol (or thiolate anion) to the cobalt(III) ion in the α-axial position (Figure 7B, step a). Then, the electron is transferred from RS^−^ to Co(III) ion, which leads to the formation of the thiyl radical and, probably, a Cbl(II)CN complex (Figure 7B, step b). The existence of a complex of Cbl(II) and CN^−^ was confirmed in [60]. Subsequent oxidation of Co(II) by dissolved oxygen results in the formation of superoxide anions and regeneration of CNCbl(III) in the system (Figure 7B, step c). 

A more detailed discussion of our results that support the proposed mechanisms of the cobalamin-catalyzed oxidation of thiols is given below.

(i) It was shown for the first time that TNB formed during the reduction of the Ellman’s reagent (DTNB) by thiols is oxidized in the presence of cobalamins (CNCbl and H_2_OCbl^+^/HOCbl). In particular, it was found that the addition of DTNB to a mixture containing NAC and cobalamin leads to a fast formation of TNB, which is subsequently oxidized by cobalamins. In addition, it was shown that cobalamins catalyze the oxidation of TNB preliminarily formed in the reaction of DTNB with DTT. The rate of oxidation of TNB by H_2_OCbl^+^/HOCbl was lower in the first case (the system containing NAC) than in the second case. In contrast, the rate of oxidation of TNB by CNCbl was somewhat higher in the first case (the system containing NAC). It is interesting that, in both systems, CNCbl catalyzed the oxidation of TNB at a 15-fold higher rate than H_2_OCbl^+^/HOCbl. These data suggest that, in the system containing H_2_OCbl^+^/HOCbl and NAC, a stable NACCbl complex is formed. The replacement of water in aquacobalamin by thiols and the formation of thiolatocobalamin complexes are evidenced by our spectrophotometric data. Based on the results obtained, it can be assumed that the slow oxidation of TNB by H_2_OCbl^+^/HOCbl in this system may be caused by the formation of the NACCbl complex, which can hinder access of thiols to the cobalt site. We also cannot exclude that the NACCbl complex itself slowly oxidizes TNB. At the same time, the rate of TNB oxidation catalyzed by CNCbl in the system containing NAC was somewhat higher, suggesting that a stable NACCbl complex does not form in this case. The reason for the higher rate of the CNCbl-catalyzed oxidation of TNB in the system containing NAC remains unclear. 

(ii) It was shown that, after a full cobalamin-catalyzed oxidation of TNB, the addition of a new aliquot of DTT results in the regeneration of TNB, after that, the oxidation of this thiol occurs over again. It was found that the rate of TNB oxidation catalyzed by H_2_OCbl^+^/HOCbl increases more than 15 times after the first round of catalysis. Probably, after coordination of the first molecule of the monothiol to the cobalt(III) ion in the β-axial position, the weakening of the Co(II)-Bzm bond takes place, which facilitates the coordination of the second molecule of the monothiol at the α-axial position. After that, the oxidation of the monothiol can occur. The molecule of TNB can coordinate at the α-axial position in such a manner that the nitro group and the carboxyl group interact with the Bzm moiety. These interactions stabilize the structure of the complex formed, leading to the inhibition of TNB oxidation. Nevertheless, monothiol is slowly oxidized to reduce the cobalt ion. The following activation of HOCbl-catalyzed oxidation of TNB remains obscure. It can be speculated that this effect is caused by an increase in the amount of the base-off form. 

(iii) According to the data of UV-vis absorption spectroscopy, during the reaction of aquacobalamin with monothiols (GSH and NAC), the formation of complexes of thiolatocobalamins occurs [11,43,48,49]. 

In contrast, no significant changes in the absorption spectrum were observed in the reaction of CNCbl with GSH or NAC. In the case of TNB, when the absorption bands of cobalamin and the thiol overlapped, spectral changes were found in the region corresponding to this thiol. No significant changes in the regions that correspond to the absorption maxima of CNCbl were observed (γ, α/β). These data indicate that, during the reaction of CNCbl with the compounds being tested, the replacement of the CN-group does not occur, suggesting that these monothiols are apparently coordinated to the cobalt ion in the α-position of the corrin ring.

(iv) DTT contains two sulfhydryl groups in the structure. As in the case of GSH and NAC, during the CNCbl-catalyzed oxidation of DTT, no significant changes in the absorption spectrum were observed, which indicates that the replacement of the CN-group does not take place. Most likely, the oxidation of dithiol DTT and monothiols being tested occurs in a similar way (Figure 7B). This is also supported by the fact that the rate of oxidation of monothiol TNB approaches the rate of oxidation of dithiol DTT.

During the reaction between dithiol and H_2_OCbl^+^/HOCbl, significant changes in absorption spectra were observed. It may be assumed that these spectral changes result from the formation of a complex of cobalamin with DTT or/and the reduction of Cbl(III) to Cbl(II). Twelve or seventeen minutes after the start of the reaction, further changes in spectra were observed. The absorbance at 465 and 312 nm decreased, whereas absorbance at 525 nm increased, which suggests the oxidation of Co(II) to Co(III) or/and the transformation of existing complexes. It should be noted that these spectroscopic changes were relatively slow. Meanwhile, the oxidation of Co(II) to Co(III) is fast enough. The second-order rate constant for this reaction is 85 M^−1^ × s^−1^ [61]. Thus, slow spectroscopic changes observed herein may be considered to be in favor of the presence of the complex of DTT with cobalamin in the system.

Taking into account that the oxidation of DTT in the presence of H_2_OCbl^+^/HOCbl was faster than the oxidation of monothiols and the spectral changes of the solutions containing DTT and H_2_OCbl^+^/HOCbl were observed in the presence of significant excess of dithiol, it can be suggested that the oxidation of DTT by aquacobalamin proceeds as follows: after the replacement of H_2_O by a thiolate anion (Figure 7A, step a), intramolecular transfer of the electron from RS^−^ to Co(III) ion occurs (Figure 7A, step b). After that, due to the presence of a second sulfhydryl group in the structure of DTT, a rapid intramolecular cyclization of dithiol proceeds (Figure 7A, step c). Then, the release of the anion radical (Figure 7A, step d), the oxidation of cobalamin (II) by dissolved oxygen, and the reformation of cob(III)alamin (Figure 7A, step e) take place. 

DDC and AA were also oxidized in the presence of cobalamins. An analysis of UV-Vis spectral changes indicated that, during the oxidation of these compounds, the replacement of the CN-group in CNCbl does not occur. Meanwhile, in the reaction between H_2_OCbl^+^/HOCbl and DDC, the replacement of the β-axial ligand in H_2_OCbl^+^ by sulfur-containing compounds takes place. We have previously shown that disulfiram and its oxidation forms, sulfones and sulfoxides, are found among the products of DDC oxidation catalyzed by H_2_OCbl^+^/HOCbl [62]. It can be assumed that H_2_OCbl^+^/HOCbl can form complexes with these derivatives. In the course of oxidation of AA catalyzed by H_2_OCbl^+^/HOCbl, significant spectral changes were observed in the region that corresponds to AA. However, difference absorption spectra showed minor changes in the regions that correspond to the absorption maxima of H_2_OCbl^+^ (γ, α/β). Thus, it can be suggested that oxidation of AA proceeds through the formation of the unstable intermediate aquacobalamin-ascorbate.

It should be noted that the proposed mechanisms do not allow one to fully explain the difference in the levels of ROS formed during thiol oxidation catalyzed by H_2_OCbl^+^/HOCbl and CNCbl.

### 3.2. Differences in ROS Production during the Oxidation of the Compounds Catalyzed by H_2_OCbl^+^/HOCbl and CNCbl

As was mentioned above, the oxidation of GSH or NAC in the presence of H_2_OCbl^+^/HOCbl led to lower CL responses than the oxidation of these monothiols in the presence of CNCbl. Meanwhile, the rate of their oxidation was higher in the presence of H_2_OCbl^+^/HOCbl. This effect may be due to the antioxidant properties of thiolatocob(II)alamin complexes formed during the oxidation of monothiols by H_2_OCbl^+^/HOCbl. Probably, thiolatocob(II)alamins reduce superoxide anions to hydrogen peroxide, which can be also reduced by these complexes. In the course of these reactions, the regeneration of thiolatocob(III)alamins takes place (Figure 7A, step h), which results in the closure of the catalytic cycle. This suggestion is supported by literature data, according to which cob(II)alamin reduces both O_2_^•−^ and H_2_O_2_ [46,63]. The rate of the oxidation of cob(II)alamin by O_2_^•−^ is close to the rate of the O_2_^•−^ dismutation by superoxide dismutase (SOD) (7 × 10^8^ versus 2 × 10^9^ M^−1^ × s^−1^) [46]. Thus, cob(II)alamin may play an important role in the elimination of ROS formed in biological systems. High antioxidant activity of glutathionylcobalamin (GSCbl) and N-acetyl-L-cysteinylcobalamin (NACCbl) has also been reported in the literature [36]. In particular, it was shown that the thiolatocobalamins protect Sk-Hep-1 cells against H_2_O_2_-induced damage. These complexes are more efficient than thiols by themselves or in combination with standard cobalamin derivatives (CNCbl, HOCbl, and MeCbl) [36]. In addition, it was proposed that glutathionylcob(II)alamin formed during the functioning of CblC reduces O_2_^•−^ to H_2_O_2_ [9,64].

In the course of the CNCbl-catalyzed oxidation of monothiols, complexes capable of scavenging ROS did not form. As a result, the integral CL-response, which reflects the total production of ROS in the system, was higher during CNCbl-catalyzed oxidation of the monothiols. The oxidation of the monothiol TNB catalyzed by CNCbl led to a high CL response, whereas during its oxidation in the presence of H_2_OCbl^+^/HOCbl, CL was not observed. In contrast to GSH and NAC, TNB in the presence of H_2_OCbl^+^/HOCbl was oxidized slower than in the presence of CNCbl. This may be the chief cause of the lower amount of ROS formed in the system containing H_2_OCbl^+^/HOCbl. Probably, the subsequent reaction of ROS with thiol and thiolatocob(II)alamin led to the elimination of ROS. As a result, no CL was observed. 

The presence of stable complexes of thiolatocobalamins capable of eliminating ROS during the oxidation of monothiols supports the assumption that the second molecule of the monothiol is coordinated to the α-axial position in aquacobalamin and is subsequently oxidized. 

The oxidation of DTT catalyzed by H_2_OCbl^+^/HOCbl led to a higher CL response than the oxidation of this dithiol catalyzed by CNCbl. The rate of DTT oxidation was also higher in the presence of H_2_OCbl^+^/HOCbl. Probably, the kinetics of this redox reaction are a decisive factor that determines the accumulation of ROS in the systems under study. In contrast, ROS are not eliminated by the DTT–cobalamin complex, which leads to their accumulation in the system.

AA induced CL only when combined with H_2_OCbl^+^/HOCbl. It can be suggested that the absence of CL in the system containing CNCbl is due to the slow oxidation of this compound (Figure S9). Most likely, ROS formed in the system react with ascorbic acid. As a result, the elimination of ROS takes place and CL is not observed. The absence of CL in a system containing any of the two vitamin B12 forms and DDC may be due to the absence of ROS production during oxidation of DDC, the antioxidant action of DDC, and/or the rapid transformation of ROS to reactive sulfur species.

### 3.3. Reactive Oxygen Species and Their Cytotoxicity Induced by Combinations of Thiols with H_2_OCbl^+^/HOCbl and CNCbl

Our previous results have indicated that combinations of HOCbl with GSH, NAC, AA [31], DTT [32], and DDC [38,39,62] produce a cytotoxic effect on HEp-2 cells. It was shown using an oxygen electrode that the oxidation of thiols [31,32] and AA [31] catalyzed by H_2_OCbl^+^/HOCbl is accompanied by the accumulation of hydrogen peroxide in culture medium. Catalase added simultaneously with these combinations completely prevented H_2_O_2_ accumulation and cell death, indicating that H_2_O_2_ plays a key role in their cytotoxic activity. Taking into account the differences in ROS production during the oxidation of the compounds catalyzed by H_2_OCbl^+^/HOCbl and CNCbl, it was interesting to compare the cytotoxic effects of these compounds in combination with cobalamins (H_2_OCbl^+^/HOCbl and CNCbl). 

It was shown that monothiols GSH, NAC, and TNB in combination with CNCbl exhibited a stronger cytotoxic effect than in combination with H_2_OCbl^+^/HOCbl. In the presence of H_2_OCbl^+^/HOCbl, dithiol DTT produced a higher cytotoxic effect than the monothiols. The data on the cytotoxicity of these combinations are consistent with the data on ROS production detected by the method of LCL during the oxidation of these thiols in the presence of corresponding cobalamins. Thus, the results obtained suggest that the combination of cobalamins with thiols induces oxidative stress, which leads to cell death. Catalase completely prevented the cell death induced by these combinations.

No significant differences in cytotoxicity between the combinations of DTT with CNCbl or H_2_OCbl^+^/HOCbl were observed, although ROS production detected by the method of LCL was higher during the oxidation of this dithiol in the presence of H_2_OCbl^+^/HOCbl. However, in both cases the production of ROS was relatively high and exceeded the production of ROS during the oxidation of monothiols by H_2_OCbl^+^/HOCbl. It can be assumed that the amount of ROS formed in both systems is sufficient to trigger processes leading to cell death. This could also explain the equally high cytotoxicity of combinations of CNCbl with all thiols. Catalase completely prevented the cell death induced by the combinations mentioned above, which supports this assumption.

AA at the concentration used had no cytotoxic effect when administrated alone or in combination with CNCbl. However, the incubation of cells with AA combined with HOCbl caused cell death. These data are consistent with the data on ROS production during the oxidation of AA in the presence of cobalamins. Catalase also completely prevented the cell death induced by the combination of AA with H_2_OCbl^+^/HOCbl. 

DDC at the concentration used was slightly cytotoxic when used alone and had a strong cytotoxic effect when added in combination with H_2_OCbl^+^/HOCbl. In contrast, no significant differences in cytotoxicity between DDC used alone and DDC in combination with CNCbl were observed. It is worth noting that ROS production was not detected by the method of LCL during the oxidation of DDC in the presence of both CNCbl and H_2_OCbl^+^/HOCbl. No hydrogen peroxide accumulation was observed using an oxygen electrode during the oxidation of DDC catalyzed by H_2_OCbl^+^/HOCbl [62]. As was mentioned above, the main products of the reaction of DDC with HOCbl are disulfiram and its oxidation forms, sulfones and sulfoxides [62]. Probably, ROS formed during the oxidation of DDC and rapidly transformed to reactive sulfur species. This could explain a partial inhibition of the cytotoxic effect of the combination of DDC with H_2_OCbl^+^/HOCbl by catalase, which was observed here and earlier [39,62]. The absence of any influence of CNCbl on the DDC-induced cell death as well as the absence of CL during the oxidation of DDC in the presence of CNCbl may be due to the slow oxidation of this compound.

The cytotoxic effect of monothiols GSH, NAC, and TNB in combination with H_2_OCbl^+^/HOCbl is worthy of special attention. It was found that the combination of GSH with H_2_OCbl^+^/HOCbl caused the lowest cytotoxicity, whereas the oxidation of this thiol in the presence of H_2_OCbl^+^/HOCbl resulted in the highest ROS production among monotiols. A combination of NAC with H_2_OCbl^+^/HOCbl produced the highest cytotoxic effect, although the oxidation of NAC in the presence of H_2_OCbl^+^/HOCbl led to a moderate production of ROS. A combination of TNB with H_2_OCbl^+^/HOCbl exhibited a moderate cytotoxic effect. However, during the first cycle of TNB oxidation in the presence of H_2_OCbl^+^/HOCbl, ROS production was not detected.

The difference in the redox behavior of different forms of vitamin B12 may underlie their different efficacy in treating cobalamin deficiency caused by defects in *cblC*. In these cases, CNCbl is inefficient [21,22]. At the same time, patients respond to high doses of HOCbl [21,22,23,24,25,26]. Probably, due to the lability of the β-axial ligand in H_2_OCbl^+^(HOCbl), a more direct precursor of the coenzyme forms may form. This could partially rescue the cofactor function of this form of cobalamin [1]. It is worth nothing that [Co^2+^]Cbl [1] and thiol-cobalamin adducts including GSCbl [18] are considered as such a precursor. One more important point should be noted. The oxidation of thiols by cobalamins may contribute to the development of oxidative stress associated with *cblC* disease. Thiolatocobalamins are considered as a potential alternative to HOCbl to treat patients with this disease [11,35]. Our data suggest that thiolatocobalamins are able to maintain the oxidation of thiols accompanied by ROS production.

## 4. Materials and Methods

### 4.1. Chemicals

Horseradish peroxidase (HRP), GSH, NAC, DTT, AA, 5-amino-2,3-dihydro-1,4-phthalazinedione (luminol), HOCbl, CNCbl, and 5,5′-dithio-bis-(2-nitrobenzoic acid) (Ellman’s reagent, DTNB) were obtained from Sigma-Aldrich (St. Louis, MO, USA). Fetal bovine serum was from Gibco (Carlsbad, CA, USA). DDC was purchased from MP Biomedicals (Irvine, CA, USA). Phosphate buffered saline (PBS) was obtained from Paneco (Moscow, Russia). All reagents were of analytical grade purity. Water used for the preparation of solutions was purified USING a Milli-Q system (Millipore, Burlington, MA, USA).

### 4.2. Cell Culture

MCF-7 cells were obtained from the Russian Cell Culture Collection (Institute of Cytology, Russian Academy of Sciences, St. Petersburg, Russia). Cells were grown in DMEM (#5648, Sigma, Missouri, MO, USA) supplemented with 10% FBS (Gibco, USA), 80 mg/L of gentamycin, and 20 mM sodium bicarbonate at 37 °C in a humidified atmosphere with 5% CO_2_.

### 4.3. Chemiluminescence Measurements

To estimate ROS production during the oxidation of the compounds in the presence of different forms of vitamin B12, the method of luminol-dependent chemiluminescence (LCL) was used for the first time. Briefly, a reaction mixture contained NaH_2_PO_4_/NaOH buffer (20 mM, pH 7.2), luminol (5 µM), HRP (1.25 U), cobalamin (25 μM), and the compounds being tested (GSH, NAC, DTT, DDC, TNB, AA) at different concentrations (15.6–1000 μM). A stock solution of 2-nitro-5-thiobenzoate (TNB) (10 mM) was prepared by adding a stoichiometic amount of DTNB to DTT in PBS (pH 7.2). Cobalamin (HOCbl or CNCbl) was added to the solutions immediately prior to the registration of the signal. LCL was recorded in 96-well plates (Greiner, Kremsmunster, Austria) at 37 °C using a multi-plate reader (Infinite 200 Tecan, Grodig, Austria). The integral LCL response was calculated as a sum of LCL values recorded during the measurements. 

### 4.4. Kinetic Studies

#### 4.4.1. Reactions of Cobalamins with GSH, NAC, and DTT

The concentration of thiols was determined using the Ellman’s reagent (DTNB). Briefly, solutions containing NaH_2_PO_4_/NaOH buffer (20 mM, pH 7.2), cobalamin (25 µM), and thiol (GSH, NAC, DTT) (100 μM) were incubated at 37 °C for different lengths of time (0, 5, 10, 20, 30, 40, 50, 60, 90, 120, 240, 360 min). Then, 2 μL of DTNB (10 mM) dissolved in DMSO was added to 100 μL of a mixture. TNB, a colored product formed in the reaction of DTNB with thiols, was detected at 412 nm. The concentration of thiols was evaluated by a calibration curve. Measurements were carried out on a multi-plate reader (Infinite F200 Tecan, Austria) in 96-well plates (Greiner, Austria).

#### 4.4.2. Reaction of Cobalamins with TNB

The oxidation of TNB in the presence of HOCbl and CNCbl was studied under different conditions. 

In the first case, a reaction mixture contained NaH_2_PO_4_/NaOH buffer (20 mM, pH 7.2), cobalamin (25 μM), and NAC (100 μM). The solution was incubated at 37 °C for 5 min. After that, 2 μL of DTNB (10 mM) dissolved in DMSO was added to 100 μL of the mixture. TNB formed in the reaction between DTNB and NAC was detected at 412 nm. The measurements were performed until the absorbance returned to the baseline. This made it possible to monitor changes in the TNB concentration with time in the presence of cobalamins. The concentration of TNB was evaluated by a calibration curve. Measurements were carried out on a multi-plate reader (Infinite F200 Tecan) in 96-well plates (Greiner).

In the second case, a reaction mixture contained NaH_2_PO_4_/NaOH buffer (20 mM, pH 7.2), cobalamin (25 µM), and TNB (100 µM). A stock solution of TNB (10 mM) was prepared by adding a stoichiometic amount of DTNB to DTT in PBS (pH 7.2). TNB was added to the solution immediately prior to the measurements. The absorbance at 412 nm, which corresponds to the absorption maximum of TNB, was recorded until absorbance values returned to the baseline (the catalytic cycle of TNB oxidation). After that, to reform TNB, 1 μL of 5 mM DTT was added to 100 μL of the mixture and the absorbance at 412 nm was further recorded. The measurements were performed during four catalytic cycles. The concentration of TNB was evaluated by a calibration curve. Measurements were carried out on a multi-plate reader (Infinite F200 Tecan) in 96-well plates (Greiner) at 37 °C.

#### 4.4.3. Reactions of Cobalamins with DDC and AA

Changes in the concentrations of AA and DDC in the presence of cobalamins were assessed by absorption spectrophotometry. Briefly, a reaction mixture contained NaH_2_PO_4_/NaOH buffer (20 mM, pH 7.2), cobalamin (25 µM), and DDC (100 µM) or AA (100 µM). The UV-Vis spectra were recorded at 37 °C every 5 min for four hours. Changes in absorption at 258 and 265 nm were used to determine changes in the concentrations of DDC and AA, respectively.

#### 4.4.4. Determination of Rate Constants 

The concentration of compounds was plotted versus the time. Pseudo-first-order rate constants were determined by a nonlinear least squares curve fit using the QtiPot 0.9.8.9 (Copyright 2004–2011 Ion Vasilief) software.

### 4.5. UV-Visible Spectral Studies

UV–Vis spectra were recorded in a 1 cm quartz cuvette on a Cary100 Scan spectrophotometer (Varian, Australia) at room temperature. A reaction mixture contained NaH_2_PO_4_/NaOH buffer (20 mM, pH 7.2), cobalamin (50 µM), as well as a sulfur-containing compound (100 µM) or AA (100 µM). To detect any spectral changes during the reaction between cobalamins and DTT, the final concentration of DTT was increased up to 500 µM. The spectra were recorded every 5 min as long as significant changes were observed.

### 4.6. Cytotoxicity Assay

Cells were seeded in 96-well microplates (Corning, NY, USA) at an amount of 10^4^ cells in 100 µL per well and incubated for 24 h. After that, cobalamins and a compound under test (GSH, NAC, DTT, TNB, DDC, AA) were added to the cells. The concentration of cobalamins in the mixture was 25 μM, and the concentration of the compounds was 0.01, 0.05, 0.10, 0.25, 0.50, 1.0, 2.5, 5, or 10 mM. The cells were incubated with added substances for 48 h. After incubation, the number of viable cells was estimated using the crystal violet cytotoxicity assay based on the staining by this dye of cells attached to a culture plate [65]. The absorbance at 620 nm was measured using a multi-plate reader (Infinite F200, Tecan, Austria). The trypan blue exclusion test was used to evaluate the number of live and dead cells in a culture.

### 4.7. Statistical Analysis

Chemiluminescence, Kinetic, and UV-Visible spectral studies: Each experiment was performed at least five times. All the values represent the means ± standard deviation. The statistical significance of the results was analyzed using the Student’s test for paired experiments, and the values of *p* < 0.05 were considered statistically significant.

Cytotoxicity assay: All experiments were performed at least three times, in triplicate. The data are given as the mean ± standard deviation. Differences between groups of data were analyzed by one-way analysis of variance (ANOVA) followed by Tukeyʹs test (GraphPad Prism 9.0.0, GraphPad Software Inc., San Diego, CA, USA). Differences with a *p* value < 0.05 were considered statistically significant.

## 5. Conclusions

To date, there is substantial evidence that cobalamins can both suppress and promote oxidative stress; however, the mechanisms underlying these effects are poorly understood. It also remains unclear how different forms of vitamin B12 affect the development/suppression of oxidative stress. Here, we demonstrate that, under certain conditions, the cobalamin-catalyzed oxidation of thiols leads to ROS production and cell death. These processes depend on the form of cobalamin and the structure of thiol. The mechanisms and kinetics of thiol oxidation by HOCbl and CNCbl differ substantially. In contrast to CNCbl, HOCbl forms stable complexes with monothiols, which exhibit high antioxidant activity and, probably, are capable of eliminating ROS formed in biological systems. This could explain higher levels of ROS production and cytotoxicity induced by combinations of monothiols with CNCbl. A complex formed between HOCbl and DTT is unstable and rapidly decomposes. As a result, ROS are not eliminated by the complex, which leads to their accumulation in the system and cell death.

On the whole, the data obtained in the frame of the present work provide a new insight into the processes in which cobalamins are involved in health and disease and might be helpful in developing new approaches to the treatment of some cobalamin-responsive disorders, in which oxidative stress is an important component.

## Figures and Tables

**Table 1:** Pseudo-first-order rate constants (k_obs_, s^−1^) for the oxidation of sulfur-containing compounds in the presence of cobalamins.

Compounds	HOCbl	CNCbl
DTT	6.33 × 10^−3^	5.14 × 10^−3^
GSH	3.99 × 10^−4^	1.53 × 10^−4^
NAC	1.47 × 10^−4^	0.75 × 10^−4^
DDC ^1^	1.70 × 10^−4^	0.50 × 10^−4^
Cycle of the oxidation of TNB		
1	6.42 × 10^−5^	1.02 × 10^−3^
2	0.99 × 10^−3^	1.33 × 10^−3^
3	1.28 × 10^−3^	1.38 × 10^−3^
4	1.89 × 10^−3^	1.40 × 10^−3^

^1^ Pseudo-first-order rate constants were calculated using UV-Vis spectra recorded during the reactions between cobalamins and DDC.

## Data Availability

Data are available within the article and Supplementary Materials.

## Supplementary Materials

The following supporting information can be downloaded at: https://www.mdpi.com/article/10.3390/ijms231911032/s1.
