# Supplementary material for: Differences in the Formation of Reactive Oxygen Species and Their Cytotoxicity between Thiols Combined with Aqua- and Cyanocobalamins
Source: Int J Mol Sci. 2022 Sep 20;23(19):11032. doi: 10.3390/ijms231911032 (PMC9569724; doi:10.3390/ijms231911032)
Supplement: Supplementary file 1 [file ijms-23-11032-s001.zip › ijms-1882346-supplementary.pdf]

International Journal of *Molecular Sciences*

MDPI

Supplementary Materials

Differences in the Formation of Reactive Oxygen Species and Their Cytotoxicity between Thiols Combined with Aqua- and Cyanocobalamins

Yuri V. Shatalin †, Victoria S. Shubina †,*, Marina E. Solovieva and Vladimir S. Akatov *

Institute of Theoretical and Experimental Biophysics, Russian Academy of Sciences, Institutskaya 3, Pushchino 142290, Russia

* Correspondence: shubinavictoria@yandex.ru (V.S.S.); akatov.vladimir@gmail.com (V.S.A.)

† These authors contributed equally to this work.

Table S1. Dependence of integral chemiluminescence response (∫LCL±SD) on the concentration of the compounds.

HOCbl (25 μM) + Reducing agent					
[Reducing agent], μM	AA	NAC	DTT	TNB	GSH
0	5008±360	4377±504	3369±288	4934±384	4934±456
15.6	5372±216	4325±528	31,657±4320#	**2977±312***	**4567±384***
31.3	**11,379±2640*#**	**4380±480***	100,763±5760#	**3034±408***	**6490±504***
62.5	**59,570±4560*#**	**4950±432***	252,490±8880#	**3020±360***	**12,064±768*#**
125	**125,013±5520*#**	**17,473±1536*#**	**539,531±7920*#**	**3130±384***	**28,783±4320*#**
250	**136,746±5760*#**	**40,352±14,160*#**	**831,704±19,680*#**	**3465±264***	**70,924±5040*#**
500	**105,146±7925*#**	42,465±7440#	**419,433±13,920*#**	**4190±264***	**44,043±11,040*#**
1000	**43,015±3782*#**	22,705±4560#	95,182±6960#	3027±432	31,535±8880#
CNCbl (25 μM) + reducing agent					
[Reducing agent], μM	AA	NAC	DTT	TNB	GSH
0	5219±240	3349±240	4150±528	4934±408	4934±384
15.6	4465±624	4965±264	48,507±5040#	**23,727±4080*#**	**55,652±3120*#**
31.3	**4559±288***	**11,628±2640*#**	126,176±5040#	**60,410±3840*#**	**95,679±2880*#**
62.5	**4567±576***	**42,791±4320*#**	257,989±8640#	**119,158±3360*#**	**147,798±4080*#**
125	**4690±336***	**146,049±13,440*#**	**266,179±26,640*#**	**147,779±3840*#**	**210,120±4320*#**
250	**4594±408***	**141,838±11,629*#**	**215,574±35,520*#**	**155,870±4800*#**	**212,621±8880*#**
500	**4742±384***	52,359±8561#	**130,578±29,520*#**	**70,343±2183*#**	**81,134±2232*#**
1000	**4716±288***	18,474±4806#	71,179±13,200#	6801±660	36,722±2040#

* significant differences between the combinations of a reducing agent with CNCbl and HOCbl (bold); # significant differences between the cobalamin-reducing agent combinations and the cobalamin (baseline), $p < 0.05$.

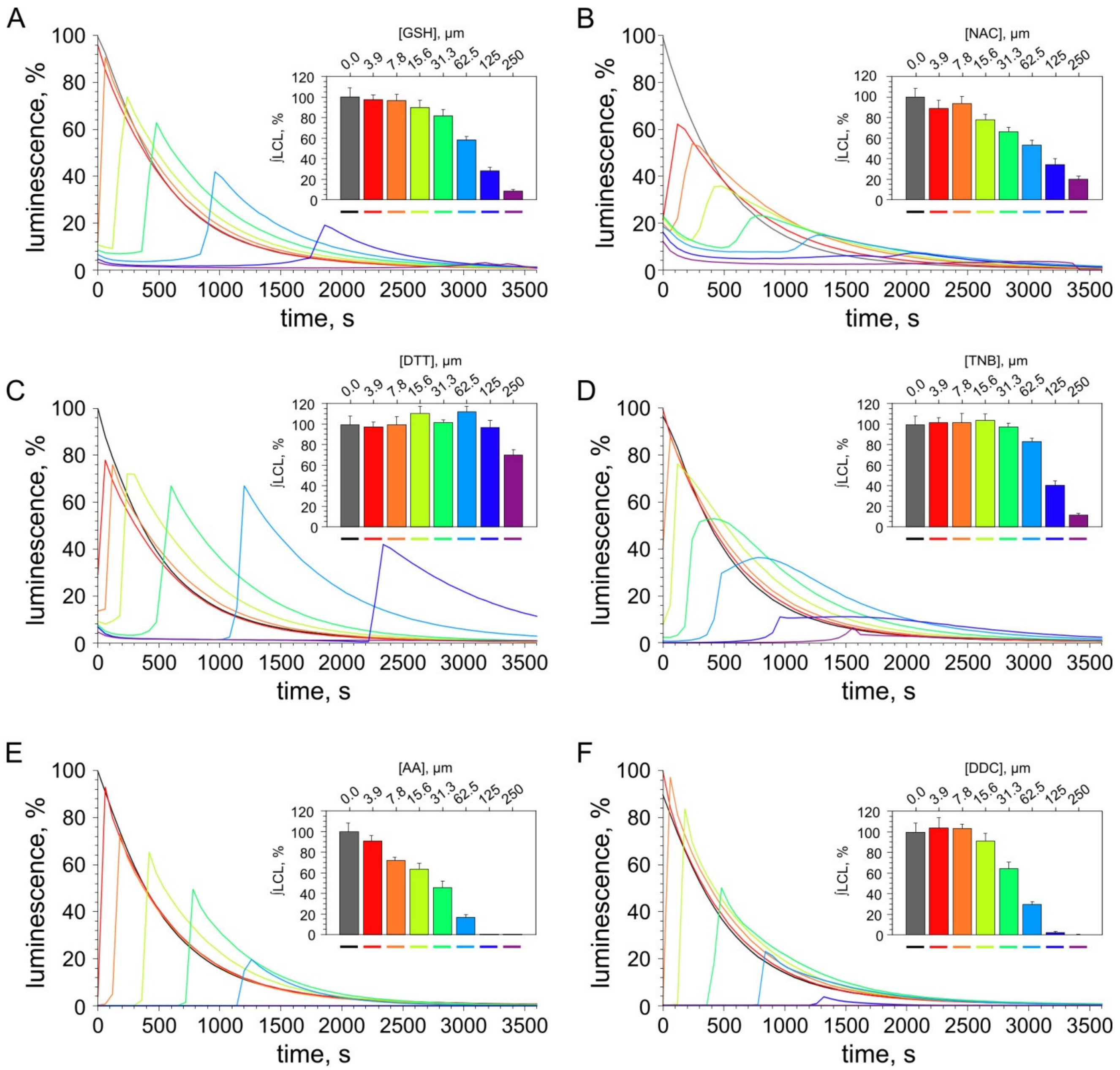

Figure S1. Effect of compounds being tested on chemiluminescence: (**A**) GSH, (**B**) NAC, (**C**) DTT, (**D**) TNB, (**E**) AA, (**F**) DDC. Inserts: Dependence of integral chemiluminescence response (∫LCL) on the concentration of the compounds.

The antioxidant activity of compounds was estimated by luminol-dependent chemiluminescence (LCL) as described previously [doi.org/10.3390/antiox10081262].

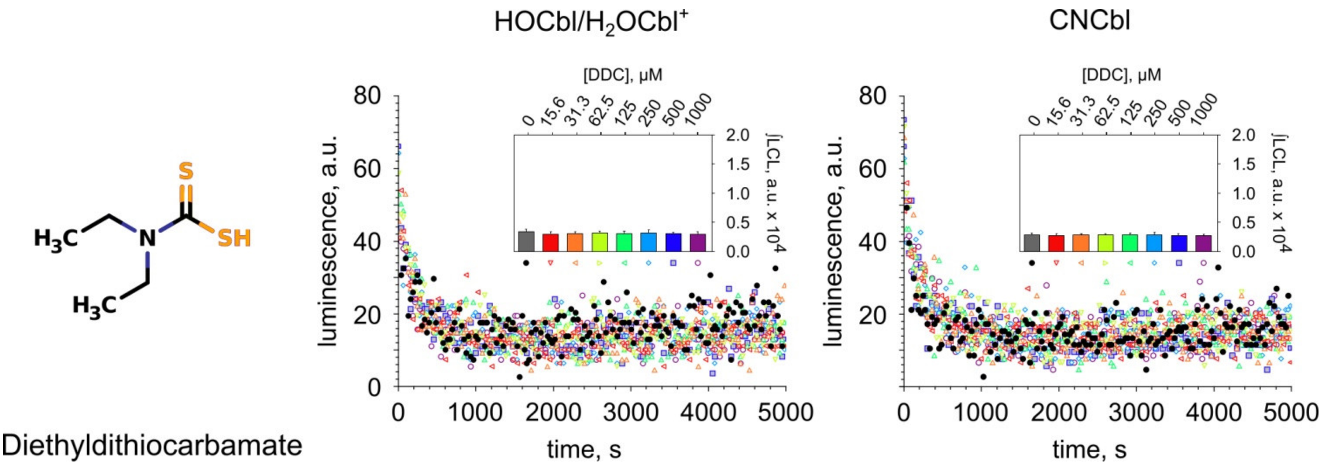

Figure S2. Chemiluminescence recorded during the oxidation of DDC in the presence of cobalamins. Inserts: Dependence of integral chemiluminescence response (∫LCL) on the concentration of DDC.

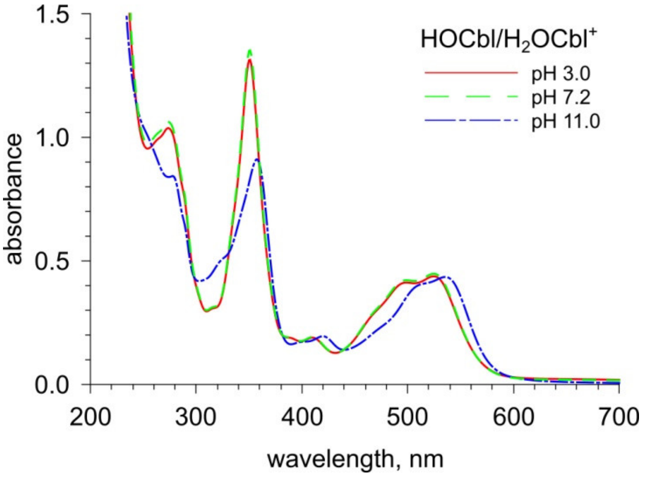

Figure S3. UV/Vis absorption spectra H_2OCbl^+/HOCbl at different pH values. The concentration of HOCbl is 50 μM.

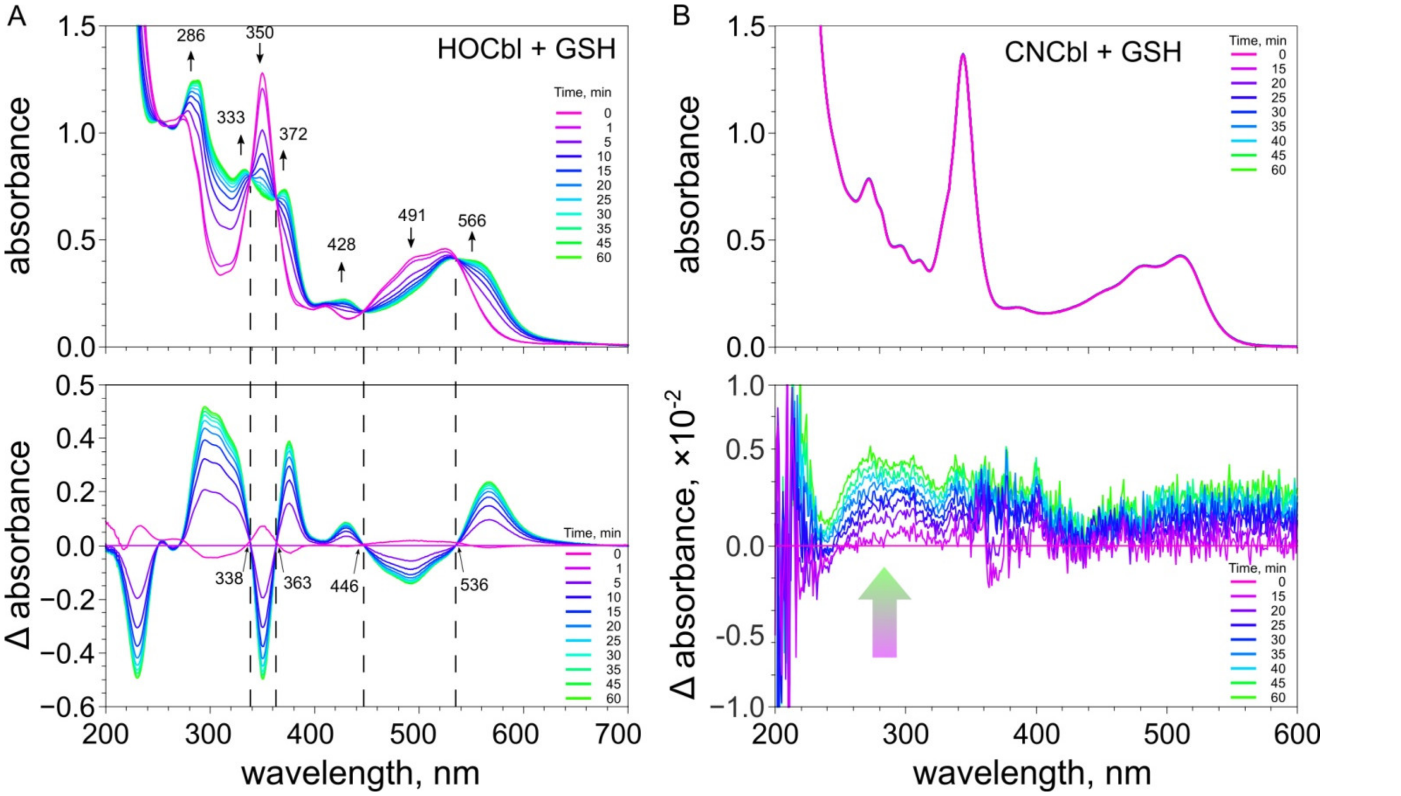

Figure S4. UV-Vis spectra recorded during the reactions between cobalamins and GSH. (**A**) - H_2OCbl^+/HOCbl, (**B**) - CNCbl. Above: absorption spectra, below: difference spectra.

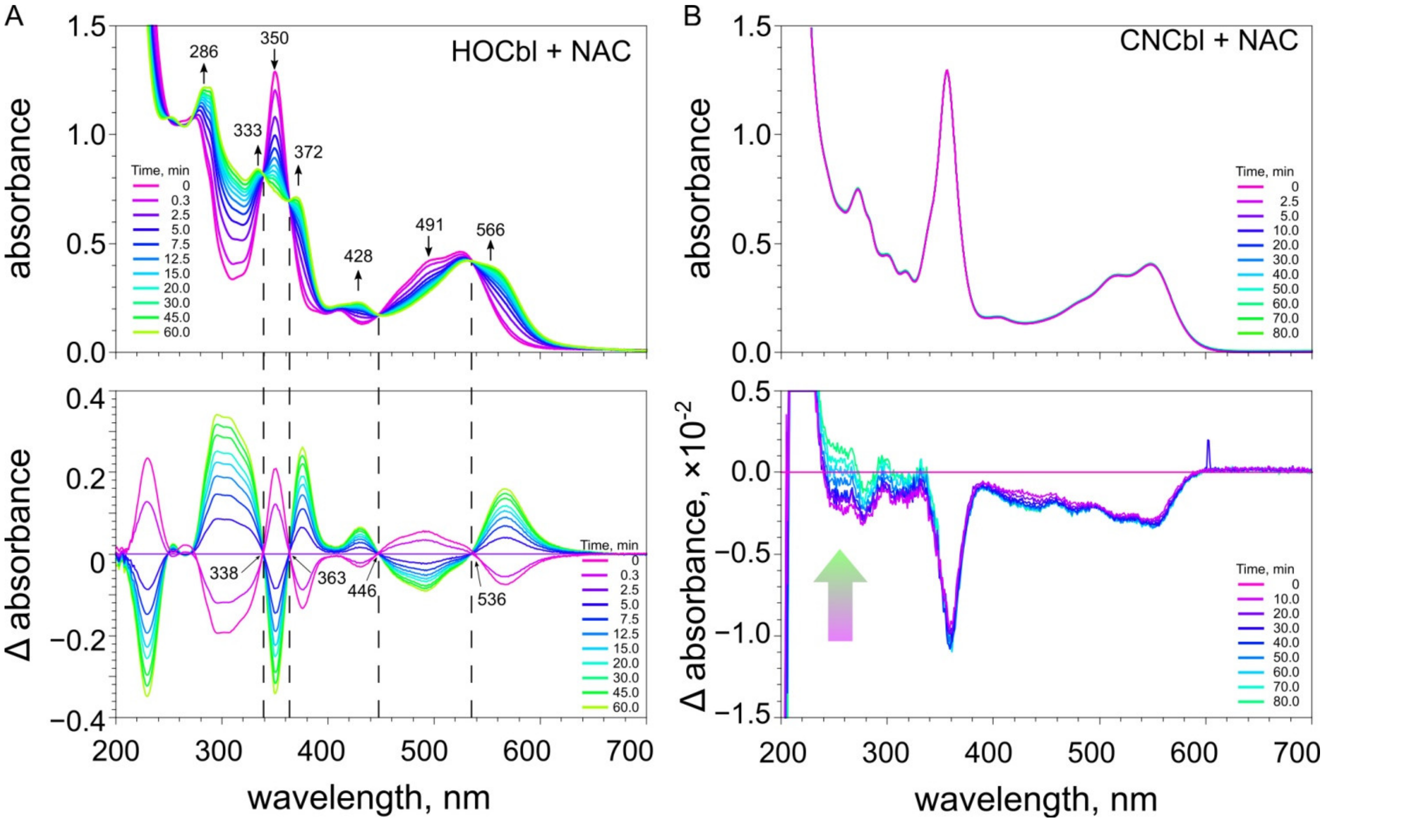

Figure S5. UV-Vis spectra recorded during the reactions between cobalamins and NAC. (**A**) - H_2OCbl^+/HOCbl. (**B**) - CNCbl. Above: absorption spectra, below: difference spectra.

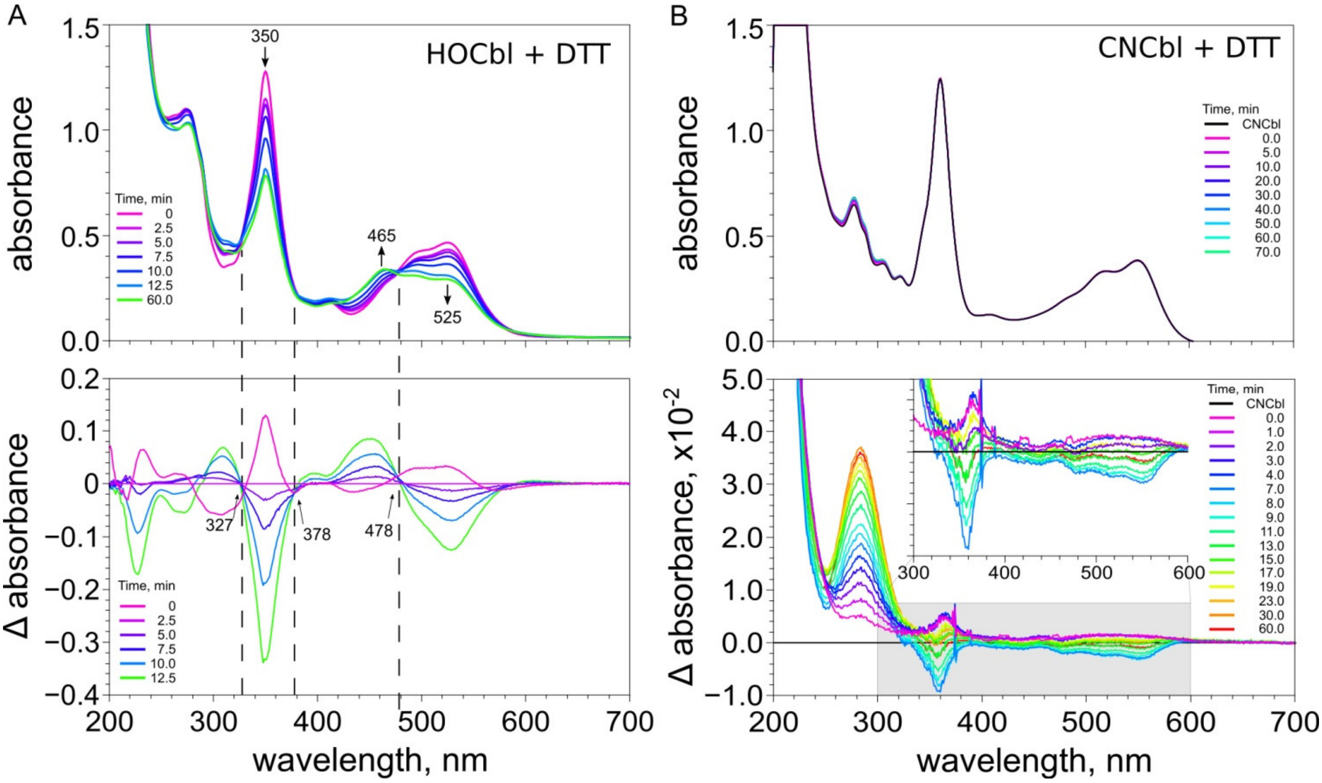

Figure S6. UV-Vis spectra recorded during the reactions between cobalamins and DTT. (**A**) - H_2OCbl^+/HOCbl, (**B**) - CNCbl. Above: absorption spectra, below: difference spectra. Inset: The difference spectra (300-600 nm) are shown in an enlarged y-axis.

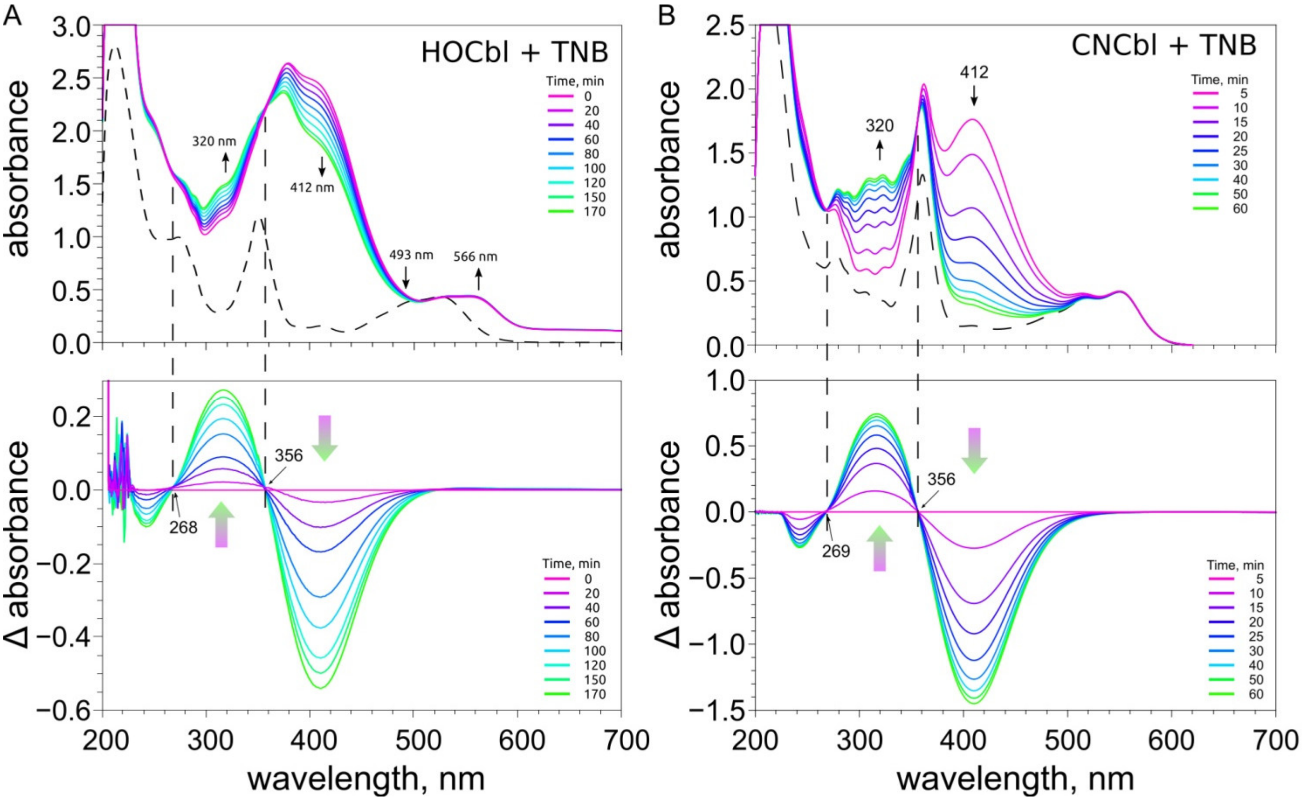

Figure S7. UV-Vis spectra recorded during the reactions between cobalamins and TNB. (**A**) - H_2OCbl^+/HOCbl. Dashed line indicates the spectrum of H_2OCbl^+/HOCbl. (**B**) - CNCbl. Dashed line indicates the spectrum of CNCbl. Above: absorption spectra, below: difference spectra.

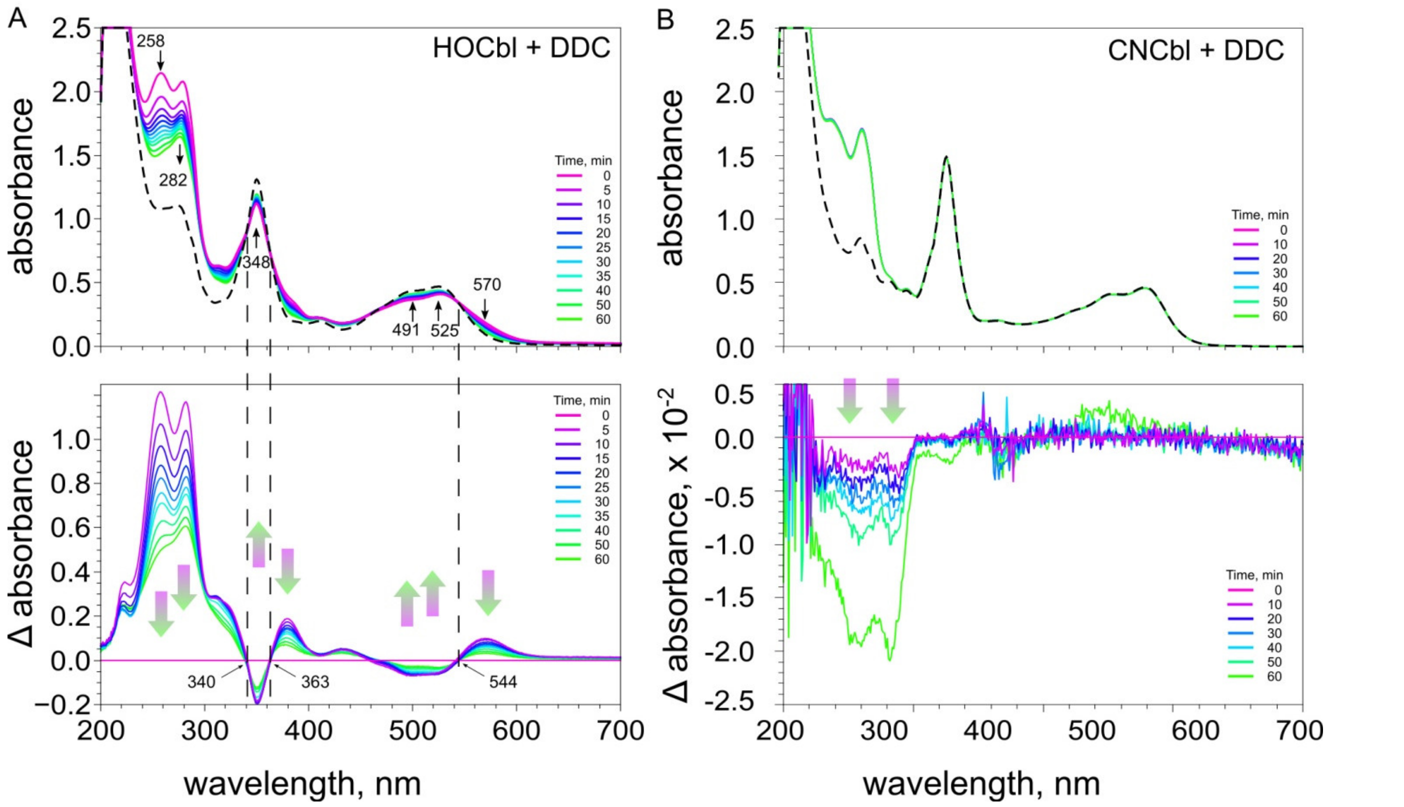

Figure S8. UV-Vis spectra recorded during the reactions between cobalamins and DDC. (**A**) - H_2OCbl^+/HOCbl. Dashed line indicates the spectrum of H_2OCbl^+/HOCbl. (**B**) - CNCbl. Dashed line indicates the spectrum of CNCbl. Above: absorption spectra, below: difference spectra.

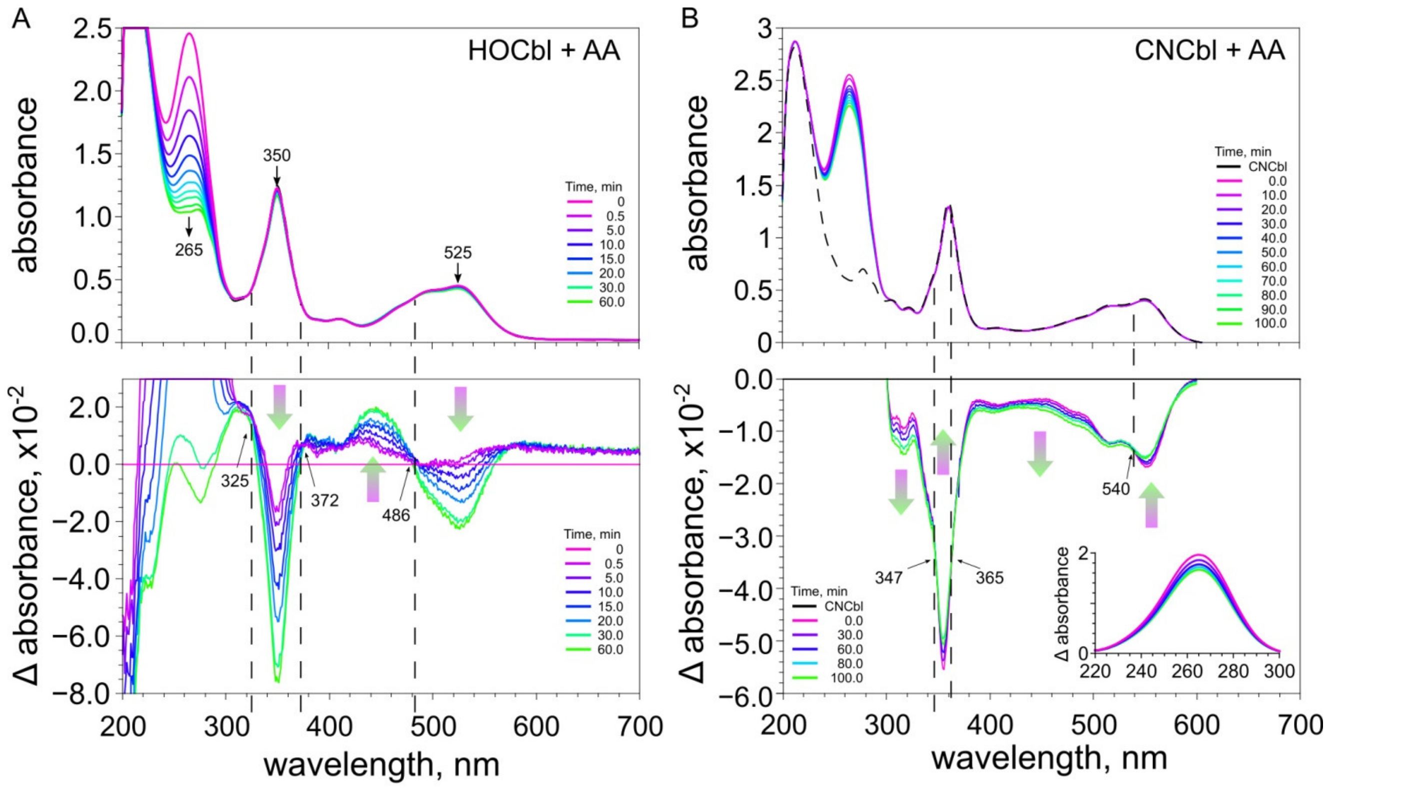

Figure S9. UV-Vis spectra recorded during the reactions between cobalamins and AA. (**A**) - H_2OCbl^+/HOCbl. Dashed line indicates the spectrum of H_2OCbl^+/HOCbl. (**B**) - CNCbl. Dashed line indicates the spectrum of CNCbl. Above: absorption spectra, below: difference spectra. Inset: The difference spectra at 220-300 nm.

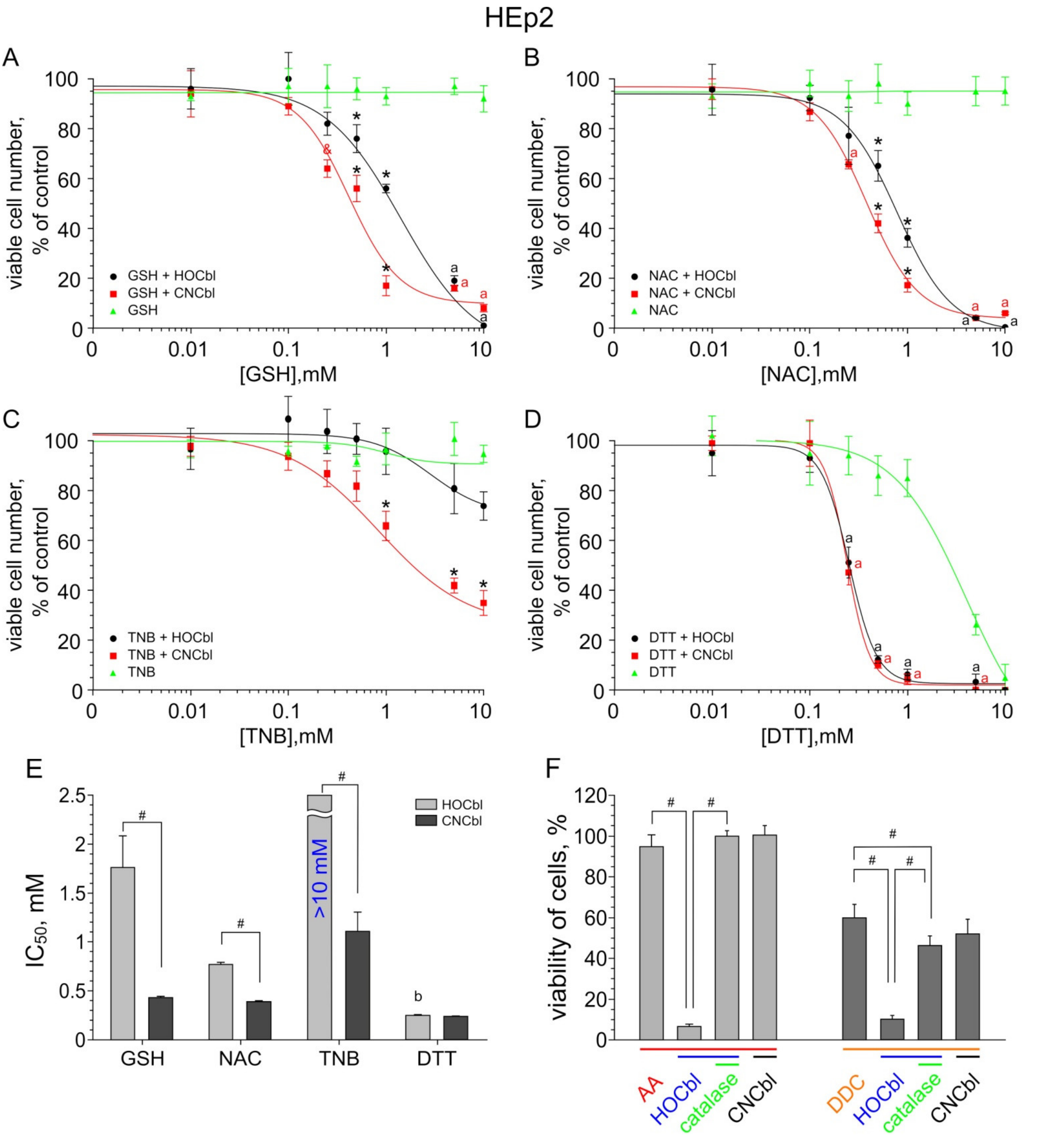

Figure S10. Cytotoxic effect of combinations of cobalamins with thiols on HEp-2 cells (**A**–**D**), IC50 values for combinations of cobalamins with thiols (**E**), and cytotoxicity of combinations of cobalamins (25 μM) with AA (0.5 mM) and DDC (1 mM) (**F**). The results were expressed as the mean ± standard deviation. Data were analyzed using one-way ANOVA followed by Tukey's test. (**A**–**D**) [*]Differences are significant compared with other two groups, $p < 0.05$; [a] significant differences between the cobalamin+thiol and thiol groups $p < 0.05$. [&] significant differences between the combinations of a thiol with CNCbl and HOCbl, $p < 0.066$ (**E**–**F**) [#] significant differences between the groups; [b] differences are significant compared with combinations HOCbl + monothiols, $p < 0.05$.
